# Acute and Chronic Toxicity of Indole Alkaloids from Leaves of *Alstonia scholaris* (L.) R. Br. in Mice and Rats

**DOI:** 10.1007/s13659-020-00237-1

**Published:** 2020-03-31

**Authors:** Yun-Li Zhao, Min Su, Jian-Hua Shang, Xia Wang, Guy Sedar Singor Njateng, Guang-Lei Bao, Jia Ma, Qing-Di Sun, Fang Yuan, Jing-Kun Wang, Xiao-Dong Luo

**Affiliations:** 1 State Key Laboratory of Phytochemistry and Plant Resources in West China, Kunming Institute of Botany, Chinese Academy of Sciences, Kunming, 650201 People’s Republic of China; 2 Yunnan Institute of Medical Material, Kunming, 650111 People’s Republic of China; 3 Key Laboratory of Medicinal Chemistry for Natural Resource, Ministry of Education and Yunnan Province, School of Chemical Science and Technology, Yunnan University, Kunming, 650091 People’s Republic of China; 4 Laboratory of Microbiology and Antimicrobial Substances, Faculty of Science, University of Dschang, P.O. Box 67, Dschang, Cameroon; 5 Jiangsu Nhwa Pharmaceutical Co., Ltd, Xuzhou, 221009 People’s Republic of China

**Keywords:** *Alstonia scholaris*, Indole alkaloids, Acute toxicity, Chronic toxicity, Non-observed-adverse-effect-level

## Abstract

**Abstract:**

*Alstonia scholaris* (L.) R. Br. (Apocynaceae) is an evergreen tree that has been used to treat lung diseases. In this study, the toxicity profile of indole alkaloids from leaves of *A. scholaris* was investigated. In acute toxicity tests, mice were administered total alkaloids (TA) and five indole alkaloids. In a chronic toxicity test, rats were continuously administered TA (50, 100, and 300 mg/kg bw) for 13 weeks, followed by a 4-week recovery. A single administration of TA affected the behavior of mice, and at 12.8 g/kg bw, prone position, shortness of breath, wheezing, and convulsion were observed. The half-lethal dose (LD_50_) in mice was 5.48 g/kg bw, almost 2740 times the clinical dose in humans. Among the five indole alkaloids, the maximum tolerance dose in mice ranged from 0.75 to 4 g/kg bw. The TA-treated rats did not die and showed no adverse effects or dose-dependent changes in weight or food and water consumption, despite fluctuations in hematological and biochemical parameters compared with historical data. Furthermore, both gross and histopathological observations revealed no abnormalities in any organ. With daily oral administration to rats, the non-observed-adverse-effect-level of TA was 100 mg/kg bw. The results indicate that TA is safe for clinical use.

**Graphic Abstract:**

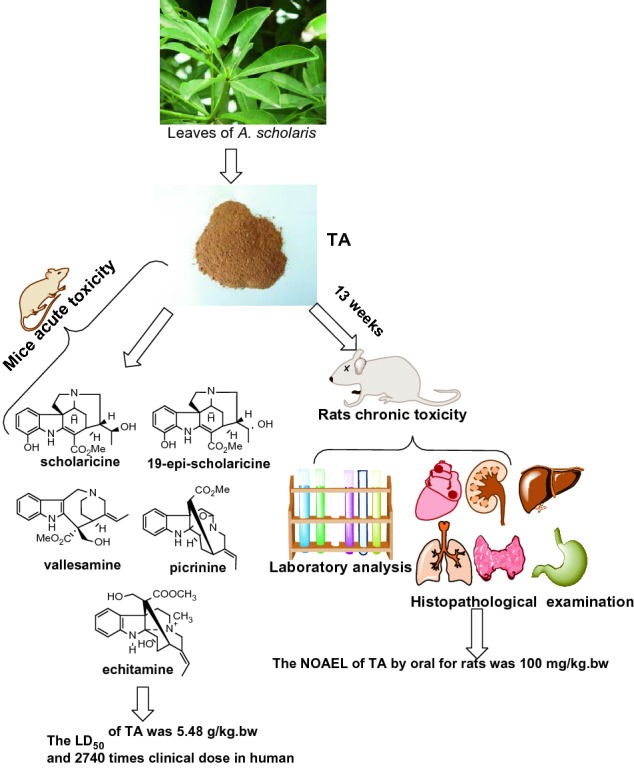

**Electronic supplementary material:**

The online version of this article (10.1007/s13659-020-00237-1) contains supplementary material, which is available to authorized users.

## Introduction

Traditional medicine has a long history of improving health worldwide, and thus, these medicines are considered to be a valuable resource for the discovery of new drugs. In recent years, the number of researchers focusing on herbal remedies has increased tremendously worldwide in order to verify or prove their efficacy after long histories of folk use. However, some products isolated from these herbal medicines have produced adverse effects. For example, aristolochic acid [1], contained in plants of *Aristolochia* and *Asarumcan*, is well known to cause kidney failure and cancer, particularly of the urinary tract in humans. Such adverse effects have aroused concern about the safety of herbal medicines [2]. Furthermore, many nonclinical studies report adverse effects or toxicities of herbal medicines after long-term use [3, 4]. Therefore, the toxicity of medicinal herbs needs to be critically assessed in order for them to be used safely by patients and clinicians.

*Alstonia scholaris* (L.) R. Br. is widely distributed in deciduous and evergreen forests and even on the plains in the tropical regions of Africa and Asia [5]. The leaves have long been used in “dai” ethno-pharmacy to treat postinfectious cough, chronic bronchitis, asthma, and other respiratory tract infections in Yunnan Province, China [6]. The authors of this paper have investigated intensively the phytochemical constituents of the different parts of the plant [7–26]. The chemical profile and metabolites of alkaloidal extract of leaves of *A. scholaris* indicate that scholaricine, 19-epischolaricine, vallesamine, and picrinine are the major indole alkaloids [27, 28].

Moreover, the extract and alkaloids of *A. scholaris* leaves have antitussive, anti-asthmatic, expectorant [29], analgesic, anti-inflammatory [30], anti-airway inflammation [31], and anti-allergic effects [32] and provide protection against postinfectious cough in vivo [33]. The alkaloids also trigger the activation of β_2_ adrenergic receptors [34] and inhibit nuclear factor-κB bioactivities in vitro [35]. Although the authors have reported on the pharmacological effects of *A. scholaris* leaves, their in vivo safety remains undetermined. Therefore, the primary purpose of this paper was to investigate the acute and chronic toxicity of the indole alkaloids of *A. scholaris* in mice and rats to help develop parameters for rational drug use.

Echitamine, a monoterpene indole alkaloid, is isolated from the stem bark of *A. scholaris.* Baliga et al. reported that the hydro-alcoholic extract of the bark of *A. scholaris* caused mortality and deformity in various organs at the dose of 240 mg/kg bw (30 days) when distributed in India, which were assumed to result from the contribution of echitamine toxicity [36]. Thus, the acute toxicity test of echitamine was also investigated in the study which suggested its safety in acute toxicity evaluation.

## Results

### Acute Toxicity

In mice in the 12.8 g/kg bw dose group, the TA produced the side effects of convulsions and death within 7–30 min after oral administration. In the 9.0 g/kg bw group, the symptoms occurred within 5–15 min and included prone position, shortness of breath, wheezing, convulsions, and death. In the other groups, the toxic effects were the same but occurred over 1–3 h, and a dose–response relationship was observed (Table 1). The dead mice were immediately dissected, and general observation revealed no changes in the size, color, and texture of organs. The collective findings were used to calculate an LD_50_ of 5.48 g/kg bw in mice, which is 2740 times the recommended clinical dose.

**Table 1:** Acute toxic symptoms of mice after the oral administration of TA

Group	Dose (g/kg bw)	Prone	Tachypnea	Whoop	Convulsion	Death
Control	–	0	0	0	0	0
TA	12.8	0	0	0	10	10
	9.0	3	1	0	6	10
	6.3	7	1	4	3	3
	4.4	2	0	1	4	5
	3.1	4	0	1	1	1
	2.2	0	0	0	0	0
LD_50_	5.48 g/kg bw					

*TA* total indole alkaloids extract, *n* the number of cases of toxic symptoms, *LD*_*50*_ half-lethal dose

In the Pic group at the dose of 2.0 g/kg bw, the activity of all mice decreased at 1 h after administration. Some animals had shortness of breath and an unstable gait. Two mice had convulsions and limb vasodilation at 3 h after administration but recovered 30 min later. These observations indicated that the maximal tolerated dose (MTD) did not exceed 2.0 g/kg bw in both sexes.

In the Val group at the dose of 4.0 g/kg bw, toxic effects were not observed during the treatment and observation periods. Thus, the MTD exceed 4.0 g/kg bw in both sexes.

In the Sch group at the dose of 0.75 g/kg bw, the activity of all mice was reduced after administration. Moreover, five mice displayed symptoms of poisoning, such as shortness of breath, unsteady gait, tremors, convulsions, and death. Therefore the MTD was less than 0.75 g/kg bw in both sexes.

In the Epi and Ech groups at the dose of 2.0 g/kg bw, no mortality or changes occurred in mice during the treatment and observation periods. Although reduced activity was observed following administration, the mice recovered 4 h later. Therefore, the MTD was 2.0 g/kg bw in both sexes.

### General Observations Associated with Chronic Oral Toxicity

The rats in each group moved freely, had shiny fur, and no deaths occurred during the study. At the 10th week of administration, sporadic alopecia was observed in one female rat in the 50 mg/kg bw group, but new hair grew gradually after ceasing the TA treatment. However, no substantial difference was detected between control and TA groups. Therefore, the alopecia in the female rat was presumed to be unrelated to the treatment with TA. No other abnormal clinical manifestations were observed.

### Food Intake, Water Consumption, and Body Weights

The food intake of female rats in all groups was not significantly affected during the administration and recovery periods (p > 0.05, Table S1). However, the food intake of male rats increased in the 50 and 100 mg/kg bw groups compared with that in the control and 300 mg/kg bw groups at the 3rd week of administration (p < 0.05, Table S1). At week 8, food intake was lower in the 50 mg/kg bw group than that in the other three groups (p < 0.05, Table S1). Overall, the changes in food intake did not show a dose–response relationship, and food intake only fluctuated slightly within the range of historical data collected in the author’s laboratory. Thus, the changes in food intake were not physiologically important and were not considered relevant to the administration of TA.

The water intake of female rats in the 50 mg/kg bw group at weeks 1 and 3 was lower than that in the 100 and 300 mg/kg bw groups (p < 0.05/0.01, Table S2). At week 5, the intake decreased in the 50 and 300 mg/kg bw groups compared with that in the control and 100 mg/kg bw groups (p < 0.05/0.01). At week 6, water intake was lower in the 50 mg/kg bw group than that in the other three groups (p < 0.05). At week 7, the intake in the 50 mg/kg bw group decreased significantly compared with that in the control and 100 mg/kg bw groups (p < 0.05), and in the 300 mg/kg bw group, it was lower than that in the 50 mg/kg bw group (p < 0.05). At week 8, a marked decrease was observed in the 50 and 300 mg/kg bw groups compared with the control and 100 mg/kg bw groups (p < 0.05). Similarly, at week 3 in male rats, the intake also decreased in the 50 mg/kg bw group compared with that in the 100 and 300 mg/kg bw groups (p < 0.05/0.01, Table S3). At week 11, water intake decreased significantly in the 50 and 100 mg/kg bw groups compared with that in the control (p < 0.01). Furthermore, the water intake in the 50 mg/kg bw group was lower than that in the 300 mg/kg bw group (p < 0.05). Thus, water intake did not show a dose–response relationship. Overall, the water intake only fluctuated slightly from normal reference values, and therefore, any changes were assumed to have no biological significance and were not considered as TA-related.

Female body weight was affected in the 300 mg/kg bw group, and the rats were not as large as those in the other three groups at week 6.5 (p < 0.01, Table S3). The body weights of males in the 50 and 300 mg/kg bw groups increased more slowly than those of the control group at weeks of 12, 12.5, and 13, and therefore, the changes in weight were not related to the dose (p < 0.05/0.01, Table S4). Overall, the changes in body weight fluctuated within the normal range of the data accumulated in the author’s laboratory, suggesting the changes were not related to TA treatment and had no clinical significance.

### Alterations in Hematological Parameters

The PT value of female rats in the 50 mg/kg bw group (12.98 ± 0.27) decreased compared with that in the other three groups in the 7 week treatment period (p < 0.05; control, 13.46 ± 0.48; 100 mg/kg bw, 14.62 ± 1.30; 300 mg/kg bw, 14.40 ± 0.92; Table S5). The WBCs increased significantly in males in the 300 mg/kg bw group (9.43 ± 1.56) compared with those in the other three groups (p < 0.05/0.01; control, 6.59 ± 1.30; 50 mg/kg bw, 6.76 ± 0.74; 100 mg/kg bw, 7.41 ± 1.56; Table S5). In addition, the WBC count (7.15 ± 1.14) in females in the 300 mg/kg bw group increased markedly compared with those of the control (WBC count, 5.24 ± 1.25) and 50 mg/kg bw groups (WBC count, 5.49 ± 1.04) after the administration of TA for 13 weeks (p < 0.01, Table S6). By contrast, the value of RDW decreased (p < 0.05; 17.18 ± 1.50 *vs.* 18.33 ± 0.76 and 18.32 ± 0.92) in the 300 mg/kg bw group. The WBC count in males in the 100 mg/kg bw group (6.56 ± 2.27) was lower than that in the control (9.13 ± 2.72) and 50 mg/kg bw (7.48 ± 1.01) groups (p < 0.05). In the recovery period (Table S7), the RBC count and Hb level of female rats in the 300 mg/kg bw group increased significantly compared with those in the other three groups (p < 0.05; RBC count, 6.78 ± 0.50 *vs.* 6.17 ± 0.30, 6.12 ± 0.34, 6.17 ± 0.29; Hb, 134.80 ± 3.03 *vs.* 124.80 ± 3.63, 127.20 ± 3.35, 125.20 ± 4.15). The percentage of monocytes (MO%) in male rats in the 50 mg/kg bw group (5.89% ± 0.23%) increased significantly compared with that in the control (4.79% ± 0.44%) and 100 mg/kg bw (4.80% ± 0.76%) groups (p < 0.01). However, all of the above changes fluctuated within the reference range of the values determined in the author’s laboratory, suggesting that the changes had no biological significance.

### Biochemical Indices

The results of the biochemical analyses from the 7-week treatment period are presented in Table S8. BUN (4.06 ± 0.51), GLU (7.54 ± 0.67), and TBIL (4.34 ± 0.22) values in females in the 300 mg/kg bw group decreased (BUN) and increased (GLU and TBIL) compared with those in the control group (p < 0.01; BUN, 5.02 ± 0.33; GLU, 6.26 ± 0.36; TBIL, 2.58 ± 1.27). Among these parameters in the 300 mg/kg bw group, the value of BUN was also lower than that in the 50 mg/kg bw group (p < 0.05; 4.90 ± 0.42) and that of GLU was also higher than that in the 50 mg/kg bw (6.42 ± 0.51) and 100 mg/kg bw (6.56 ± 0.40, p < 0.01) groups. Similarly, the values of TBIL (3.98 ± 0.84) and Cl^−^ (109.08 ± 1.25) in female rats in the 100 mg/kg bw group increased compared with those in the control group (p < 0.05; TBIL, 2.58 ± 1.27; Cl^−^, 107.44 ± 0.72). Furthermore, the value of Cl^−^ was also higher than that in the 300 mg/kg bw group (p < 0.05; 107.20 ± 1.00). However, the TC content in male rats in the 100 mg/kg bw group was lower than that in the control and 300 mg/kg bw groups (p < 0.01/0.05; 1.13 ± 0.14 vs. 1.57 ± 0.36 and 1.44 ± 0.18).

The results of the biochemical analyses from the 13-week treatment period are presented in Table S9. The contents of ALP, GLU, and Cl^−^ in females from the 300 mg/kg bw group increased significantly compared with those in the other three groups (p < 0.01; ALP, 75.20 ± 14.97 *vs.* 56.90 ± 12.62, 61.30 ± 7.12, and 61.30 ± 7.90; GLU, 8.11 ± 1.18 *vs.* 6.40 ± 0.64, 6.55 ± 0.51, and 6.74 ± 0.68; Cl^−^, 100.24 ± 0.93 *vs.* 102.59 ± 1.76, 102.06 ± 2.17, and 102.14 ± 2.20). By contrast, the content of AST decreased dramatically in the 300 mg/kg bw group compared with that in the control and 50 mg/kg bw groups (63.30 ± 9.63 *vs.* 76.70 ± 12.55 and 79.50 ± 11.85). The content in the 100 mg/kg bw group (69.50 ± 8.15) also decreased compared with that in the 50 mg/kg bw group (p < 0.05). The changes in these parameters in males were different from those in females, and the value of TG in the three TA treatment groups increased significantly compared with that in the control (*p* < 0.05; 0.99 ± 0.31, 1.04 ± 0.37, and 1.01 ± 0.26 *vs.* 0.69 ± 0.27). In addition, the ALT content in the 300 mg/kg bw group was lower than that in the control and 100 mg/kg bw groups (p < 0.05/0.01; 29.30 ± 7.24 *vs.* 36.70 ± 5.70 and 37.70 ± 4.22).

The results of the biochemical analyses from after the recovery period are shown in Table S10. The content of AST in the 100 (59.20 ± 7.01) and 300-mg/kg bw (61.00 ± 5.79) groups decreased significantly compared with that in the control group (p < 0.01; 79.40 ± 12.34) in female rats. In addition, the AST content in the 100 mg/kg bw group was lower than that in the 50 mg/kg bw group (p < 0.05, 71.80 ± 10.35). In male rats, the contents of ALP and BUN in the 300 mg/kg bw group were lower than those in the control and 50 mg/kg bw groups (ALP, 77.60 ± 9.21 *vs.* 102.00 ± 11.73 and 105.00 ± 22.59; BUN, 3.24 ± 0.17 *vs.* 4.16 ± 0.28 and 3.68 ± 0.26). In addition, the BUN value in the 300 mg/kg bw group was also lower than that in the 100 mg/kg bw group (p < 0.01, 3.98 ± 0.41). The BUN value in the 50 mg/kg bw group (3.68 ± 0.26) was also lower than that in the control group (p < 0.05, 4.16 ± 0.28).

Overall, although these indicators were abnormal, they all fluctuated within the range of values accumulated by the author’s laboratory, and no significant dose–response or time–response relationship was detected. Therefore, these changes were not considered as related to the administration of TA.

### Organ Coefficients

The organ-to-weight ratios of rats are presented in Table S11. In both females and males in the 300 mg/kg bw group, the liver coefficients increased compared with those in the other three groups in the 7-week treatment period (p < 0.05/0.01; female, 3.37 ± 0.24 *vs.* 2.69 ± 0.19, 2.78 ± 0.17, and 2.76 ± 0.18; male, 3.31 ± 0.10 *vs.* 2.91 ± 0.18, 2.79 ± 0.05, and 3.05 ± 0.22). The kidney coefficient also increased in females in the 300 mg/kg bw group compared with that in the other groups (0.70 ± 0.01 *vs.* 0.63 ± 0.04, 0.64 ± 0.04, and 0.62 ± 0.03).

After 13 weeks of treatment (Table S12), the liver index in females increased significantly (p < 0.05) in the 300 mg/kg bw group compared with that in the other three groups (3.52 ± 0.26 *vs.* 2.66 ± 0.11, 2.59 ± 0.15, and 2.72 ± 0.20); the kidney index also increased significantly in the 300 mg/kg bw group (p < 0.01; 0.62 ± 0.06*,* 0.60 ± 0.04, 0.60 ± 0.04, 0.69 ± 0.05). Similarly, the liver coefficients in males in the three TA treatment groups increased significantly compared with that in the control (p < 0.01; 2.56 ± 0.10 *vs.* 2.73 ± 0.14, 2.85 ± 0.10, and 3.31 ± 0.12; Table S12). The brain coefficients increased significantly in male rats in the 300 mg/kg bw group (p < 0.05/0.01; 0.48 ± 0.03 *vs.* 0.44 ± 0.04, 0.45 ± 0.04, and 0.45 ± 0.02). By contrast, the testis coefficients of the 50 and 100 mg/kg bw groups decreased compared with that in the 300 mg/kg bw group (p < 0.05/0.01; 0.63 ± 0.04 and 0.60 ± 0.04 *vs.* 0.67 ± 0.06). After the recovery period, no significant (p > 0.05) changes were detected in any organ coefficients (Table S13).

### Histopathological Changes

Gross necropsy revealed no adverse effects in any organ after the 13-week treatment of TA. Histopathological examinations of the main tissues from rats in the control and 300 mg/kg bw groups revealed some lesions, primarily in the liver, lung, and prostate, and their incidence was similar between the two groups (p > 0.05, Table 2, Figs. S1–S3). The histopathological changes in the livers included scattered spotty necrosis or small focal inflammation of partial hepatocytes and steatosis. Most of the lungs had widened alveolar walls and lymphocyte infiltration of the pulmonary interstitial, and prostatitis occurred in some animals. The prostatic interstitium was widened, and a small amount of inflammatory cell infiltration was composed primarily of lymphocytes and monocytes. However, no significant histological lesions were observed in the kidney, spleen, stomach, small intestine, colon, brain, adrenal gland, testis, ovary, or uterus.

**Table 2:** The abnormal histopathological findings

Time	Organ	Pathologic changes	Grading	Control		300 mg/kg bw	
				Female	Male	Female	Male
7 week	Liver	Scattered spotty necrosis or small focal inflammation	±	4	1	5	2
			+	0	1	0	0
		Hepatocyte steatosis	±	1	1	1	3
			+	1	0	0	0
	Lung	Local interstitial pneumonia	+	5	5	4	4
		Small granulomatous pneumonia	+	0	0	1	1
	Prostate	Interstitial lymphocyte infiltration	±	1	–	0	–
			+	3	–	4	–
			++	1	–	1	–
13 week	Liver	Scattered spotty necrosis or small focal inflammation	±	4	4	1	2
			+	3	1	3	3
		Hepatocyte focal necrosis	+	1	1	0	0
		Hepatocyte steatosis	±	5	2	4	0
			+	1	1	4	3
	Lung	Local interstitial pneumonia	±	0	0	0	1
			+	10	10	10	9
	Prostate	Interstitial lymphocyte infiltration	+	6	–	5	–
Recovery period	Liver	Scattered spotty necrosis or small focal inflammation	±	0	2	3	1
			+	1	2	1	1
		Hepatocyte steatosis	±	1	2	2	0
			+	2	0	0	0
	Lung	Local interstitial pneumonia	+	5	5	4	4
	Prostate	Interstitial lymphocyte infiltration	+	3	–	3	–

The specific number represented the number of abnormal animals. The total number of animals of each group was 10, 20 and 10 after the TA administration for 7, 13 weeks and 4-week recovery period (half male and half female), respectively. Results showed there was no statistically significant difference between the control group and the 300 mg/kg bw (*p* > 0.05). Animals in the 50 mg/kg bw and 300 mg/kg bw groups were not evaluated histopathologically

± The degree of lesion was slight

+ The degree of lesion was mild

++ The degree of lesion was moderate

– The item was lacking

## Discussion

The use of natural drugs is increasingly popular in healthcare and other areas worldwide. However, their safety remains a major public health problem. Because these agents are generally perceived as being harmless to the body, they are used in self-therapy without supervision. Although the bioactive compounds in medicinal plants exert a variety of beneficial biological effects in humans, knowledge of their potential toxicities is limited. The leaves of *A. scholaris* have a long history of treating whooping cough in the Dai people in Yunnan Province. Previously, the author’s group demonstrated the efficacy of the total indole alkaloid extract of *A. scholaris* leaves in respiratory management. Although the safety of the extract of stem bark of *A. scholaris* has been evaluated [36], the potential toxic effects of the indole alkaloids of leaves remain unknown. Accordingly, in the present study, the acute and chronic oral toxicity of the indole alkaloids in the leaves of *A. scholaris* was evaluated in rodents.

An acute toxicity test is used to evaluate any adverse effects appearing within a short time after a single large dose of the test substance or after multiple doses given within 24 h. In this study, oral exposure to the TA extract at a dose from 2.2 to 12.8 g/kg bw triggered important toxic responses in mice, including prone position, tachypnea, whoop, and convulsions, in a dose–response relationship. These toxic symptoms might be related to toxicity to the neuromuscular, central nervous system and autonomic nervous system. The LD_50_ value for the mice was 5.48 g/kg bw, which is approximately 2740 times higher than the recommended clinical dosage for patients (2 mg/kg/d). And the MTD was 2.2 g/kg bw, correspondingly, the doses converted into four main compounds were 220 mg/kg bw (Pic), 132 mg/kg bw (Sch), 132 mg/kg bw (Val) and 22 mg/kg bw (Epi), respectively. Of the four compounds tested, both Pic (2.0 g/kg bw) and Sch (0.75 g/kg bw) caused the same toxic responses of shortness of breath, unsteady gait, tremors, convulsions, and death. However, a toxic response was not observed in the Val and Epi groups. Under the conditions of this experiment, the MTDs were 2.0 g/kg bw (Pic), less than 0.75 g/kg bw (Sch), more than 4.0 g/kg bw (Val) and 2.0 g/kg bw (Epi). In the analysis of gross anatomy, no abnormalities were observed in any of the internal organs, including the liver and kidney, when compared with the control group. The conclusion was that the MTDs of four compounds in TA was smaller after comparing the tested result with that calculated. We speculated that there were other trace components and non-alkaloid constituents which affect the safe dose of total alkaloid extract in addition to the four major components. Next, the absorption of TA in vivo could be affected by gastrointestinal enzymes of animals, acid dissociation constant, partition coefficient (log p), absorption site (stomach-duodenum), chemical and physiological polymorphisms, cytochromes, etc.

Echitamine, a monoterpene indole alkaloid, did not cause a toxic reaction in the mice in this study and was well tolerated at the dose of 2 g/kg bw without any acute toxicity. Furthermore, echitamine has not been detected in the leaves of *A. scholaris* collected in Yunnan Province, although it was isolated from the bark in a previous investigation [37]. Thus, the good tolerance and the absence of echitamine in all tested leaves of *A. scholaris* suggested that no need to limit its quantity in a botanic drug from TA.

A chronic toxicity study is typically conducted from 1 to 3 months, because some substances that do not cause immediate toxicity may cause toxic effects after repeated exposure. According to the testing guidelines for the safety evaluation of drugs (Notification [Z] GPT3-1) issued by the China Food and Drug Administration on March 2005, the period for testing drug administration in animals should be based on the expected period of clinical use of that substance in humans. The repeated oral administration of a test substance for 1–3 months in animals is thought to be comparable to the administration for less than 1 month in humans. The objective of chronic toxicity studies is to determine the possible clinical adverse reactions caused by the substance, including the nature and degree of harm, the dose–response and time–response relationships, the effects on target organs or tissues, and the reversibility, and then predict the starting dose in clinical trials and the safe dose range for repeated drug use. The doses of 50, 100, and 300 mg/kg bw TA used in this study were equivalent to 25, 50, and 150 times, respectively, the usual dose in humans.

In this study, both male and female rats treated with doses of 50, 100, and 300 mg/kg bw TA once daily showed no signs of clinical toxicity or mortality. Changes in food and water ingestion are generally used as indicators of the harmful effects of drugs and chemicals [38]. In the experiments in this study, the two parameters were not significantly affected in weekly measurements, except for slight fluctuations in the TA treatment groups relative to the control group that were within the normal ranges of values. In addition, the alopecia of one female rat in the 50 mg/kg bw group was considered incidental given the frequencies across groups. Thus, the findings of this study suggested that different doses of TA from 50 to 300 mg/kg bw intragastrically administered to rats for 13 weeks had no significant effects on general behavior, mental state, or food intake.

The liver and kidneys are frequent targets of drug action, because the liver is the primary organ for drug biotransformation, and the kidneys are the primary organs for drug excretion. Organ weight is one of the main parameters used to evaluate the effects of test substances in toxicity studies [39]. In the present investigation, the liver and kidney organ coefficients in the 300 mg/kg bw group increased significantly in the 7th and 13th weeks compared with those in the control group. Meantime, we found the body weight showed a decreasing trend at the corresponding time. It thus was speculated the decrease of the two coefficients was induced by the decrease of body weight. However, weight loss was due to shrinkage of absorption of nutrients by the intestinal, but the slight fluctuations of food/water consumption in the TA-treated groups were within the normal ranges of values. In addition, no significant differences were observed in the serum levels of ALT, AST, CRE and BUN. Some test substances may harm tissues at the cellular level but not cause any observable abnormalities in an organ. For this reason, histopathological examination of livers and kidneys was conducted to identify any cellular damage in the internal organs or tissues. Fortunately, no significant histopathological changes in liver tissues in the 300 mg/kg bw group were observed relative to the control group. Moreover, the biochemical indicators of liver and kidney function were normal after the withdrawal of TA for 4 weeks. The above results all indicated that the increase in liver and kidney coefficients during the administration period was not toxicologically significant. In addition, Treatment-related changes of Glu, an indicator of pancreatic function [40], was not observed, signifying that the TA did not damage pancreas function.

The hematopoietic system, which is highly sensitive to toxic substances, can be altered when poisonous plants are ingested [41]. And changes in hematological parameters can reflect adverse effects on bone marrow function, such as anemia and hemolysis [42]. White blood cell counts are commonly determined to assess immune function [40]. In this study, white blood cell and red blood cell counts of the TA-treated groups were comparable to those of the control group. In addition, no significant differences in weight of the thymus were observed, an important lymphoid organ associated with the immune system [43]. The exception was the higher WBC counts in the 300 mg/kg bw male and female rats than those in the controls at the 7th and 13th weeks. The values of the other hematological parameters examined (Hb, RDW, MCV, MCH, PLT, PT, and RET) were within the ranges of normal reference values [39]. Thus, the collective findings of the study indicated no adverse effects of TA on the hematology and immune system of rats.

Histopathological examination is a fundamental step in preclinical toxicology research that is used to further validate whether tissues or internal organs are damaged [42]. According to the Organization for Economic Co-operation and Development guidelines, microscopic examination of organs is not required in the 50 mg/kg bw group in cases in which no histopathological alterations are observed in the 300 mg/kg bw group [44]. In this study, no apparent histopathological changes were observed in the main organs and tissues (heart, kidney, spleen, stomach, small intestine, colon, brain, adrenal gland, testis, ovary, uterus) of rats treated with 300 mg/kg bw TA. The exceptions, which were observed in both the control and 300 mg/kg bw groups, were small focal inflammations in livers, local interstitial pneumonia in lungs, and interstitial lymphocyte infiltration in prostates. According to previous reports [45], these types of lesions are commonly associated with Sprague–Dawley rats of the age used in this study and are considered to be related to spontaneous or iatrogenic causes. The lesions were similar in severity in all groups and were graded as either minimal or mild and were therefore not considered as TA-related. Thus, the test substance showed no accumulative toxicity when administered at doses of 50, 100, and 300 mg/kg bw TA for consecutive 13 weeks and after a 4-week recovery period following withdrawal of the TA treatment. All these results indicated that the TA and the five main compounds were safe for human use.

## Conclusions

In summary, at the tested oral doses ranging from 2.2 to 12.8 g/kg bw, TA produced some signs of acute toxicity in mice in a dose-dependent manner, which might be related to toxicity to the neuromuscular, central nervous system and autonomic nervous system. The half-lethal dose (LD_50_) (administered via gavage) in mice of 5.48 g/kg bw was approximately 2740 times the recommended dose for patients. In addition, acute toxicity testing demonstrated that picrinine, vallesamine, sholaricine, 19-epischolaricine, and echitamine were safe for mice when used alone. Moreover, no lethality, significant alterations in hematological and serum biochemical indices, or adverse histopathological effects were evident in the chronic toxicity study (13 weeks) with rats using doses 25, 50, and 150 times the clinical dose. On the basis of this result, the TA should be considered safe for use in clinics and deserve further development as a pharmaceutical product.

## Methods

### Plant Material

Leaves of *A. scholaris* were purchased from Datang–Hanfang Medicine Co., Ltd. (Pu’er, China). The plants were collected in 2006 in Pu’er City, Yunnan Province, People’s Republic of China. Dr. Xiao–Dong Luo of the Kunming Institute of Botany, Chinese Academy of Sciences, identified the plants, and the plant name was checked against https://www.theplantlist.org. A voucher specimen (no. Luo20060407) was deposited in the State Key Laboratory of Phytochemistry and Plant Resources in West China, Chinese Academy of Sciences.

### Preparation of Alkaloids

**Fig. 1:**
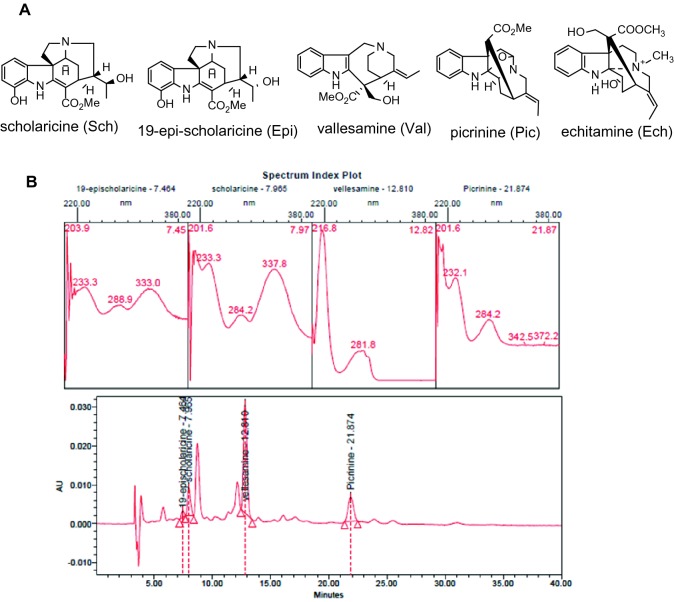
HPLC/UV chromatograms of total alkaloids (285 nm). A. Four major alkaloids from leaf and echitamine from bark of *A. scholaris.* B. HPLC/UV chromatograms of total alkaloids

Dried and powdered leaves of *A. scholaris* were extracted with 90% ethyl alcohol under reflux conditions (3 h × 4 times) at room temperature, and the solvent was evaporated in vacuo to obtain the ethanolic extract. The ethanolic extract was dissolved in 0.3% aqueous HCl solution and filtered; the residue was the nonalkaloid fraction. Then, the acidic solution, adjusted to pH 9–10 with 10% aqueous ammonia, was extracted with ethyl acetate to obtain the total alkaloids (TA) fraction (batch no. 20070512). In addition, the picrinine, vallesamine, sholaricine, 19-epischolaricine, and echitamine used in this study were isolated from the leaves or barks of *A. scholaris* (Fig. 1a) in a previous phytochemical investigation and kept in a refrigerator. The established HPLC/UV method was used to determine the four major alkaloids of the TA (See Supporting information, S1.1–1.2). The typical HPLC/UV chromatograms of TA are shown in Fig. 1b and the chromatography profile includes four high-peaks at retention time of 7.464 (19-epischolaricine), 7.965 (scholaricine) 12.810 (vallesamine) and 21.874 min (picrinine).

### Experimental Animals

The experiment was reviewed and approved by the Institutional Animal Care and Use Committee of the Yunnan Institute of Materia Medica. The tests were performed at the Yunnan Institute of Materia Medica in compliance with Good Laboratory Practice regulations established by the China Food and Drug Administration. All activities related to animal care and handling were performed according to the Guide for the Care and Use of Laboratory Animals and the policies of Association for Assessment and Accreditation of Laboratory Animal Care International.

Specific pathogen-free Sprague–Dawley (SD) rats (60–110 g) and Institute of Cancer Researcch (ICR) mice (18–20 g) were purchased from Kunming Medical University, Kunming City, Yunnan Province, People’s Republic of China (license no. SCXK (Dian) 2005-0008). Animals were housed in a room at 24 ± 1 °C and 50–60% relative humidity under a 12 h light–dark cycle and maintained with lights on at 9:00 and off at 21:00. The animals were acclimatized to the laboratory environment for 7 days and allowed free access to water and a standard diet before the initiation of treatment. All animals were fasted but provided water for 10–12 h before the start of the experiment. Animals were euthanized using pentobarbital sodium (50 and 40 mg/kg bw via intraperitoneal injection for mice and rats, respectively) followed terminal heart stick and cervical dislocation or induction of bilateral pneumothoraces as secondary means of assuring euthanasia. There are no deviations from the standards and regulations promulgated under the Animal Welfare Act.

### Acute Toxicity

Acute toxicity was evaluated in mice according to the testing guidelines for the safety evaluation of drugs (Notification [Z] GPT2-1) issued by the China Food and Drug Administration in March 2005. In the experiment, the dose of all test substances were set according to the pretest results (Data not shown). Fasted mice were randomly divided into different groups of 10 each (half male and half female). The groups were the following.

Total indole alkaloids (TA): The mice were divided into seven groups and treated with TA orally at doses of 0, 2.2, 3.1, 4.4, 6.3, 9.0, and 12.8 g/kg bw. The treated groups were administered TA at the volume of 30 mL/kg, whereas the control group (0 g/kg bw) received an equal volume of 1% carboxymethylcellulose.

Alkaloid compounds: Five groups were intragastrically administered picrinine (Pic) at 2.0 g/kg bw, vallesamine (Val) at 4.0 g/kg bw, scholaricine (Sch) at 0.75 g/kg bw, 19-epischolaricine (Epi) at 2.0 g/kg bw, or echitamine (Ech) at 2.0 g/kg bw. The treated groups were administered the different compounds from *A. scholaris* at the volume of 20 mL/kg, whereas the control group received an equal volume of 1% carboxymethylcellulose.

The symptoms of toxicity and mortality were recorded systematically at 1, 2, 4, and 6 h after administration of the test substances. The number of live mice was noted after 24 h, and once-daily observations were performed for a further 14 days. The animals were intraperitoneally injected with pentobarbital sodium and sacrificed by cervical dislocation to gross observation.

### Chronic Toxicity

A chronic toxicity test was performed on rats in order to evaluate the target organs and the reversibility of *A. scholaris* adverse effects according to the testing guidelines for the safety evaluation of drugs (Notification [Z] GPT3-1) issued by the China Food and Drug Administration on March 2005. Treatments were administered for 13 weeks, followed by 4 weeks of recovery. In this test, 160 rats were divided into four groups of 40 rats (20 female and 20 male) that were treated orally with 0 (control), 50, 100, or 300 mg/kg bw TA in a volume of 10 mL/kg daily. Rats in the control group were given an equal volume of 1% carboxymethylcellulose. Daily observations were conducted for toxicological signs, mortality, and physiological and behavioral changes. Bodyweights were recorded on day one of the study and twice weekly thereafter. Body alterations were recorded, and the dose was adjusted in accordance with body weight to maintain the target dose level in all animals. Food consumption and water intake were also measured each week. Rats were anesthetized and blood samples were collected from the common carotid artery with EDTA-2K, sodium citrate (1:9), vacuum blood collection tubes. The hematological, coagulative, and biochemical indices were evaluated (see methodology below) after TA treatment for 7 and 13 weeks and after the 4-week recovery period. Following blood collection, gross observation of the major organs was carefully performed, and the absolute and relative (organ to body weight ratio) weights of organs and tissues, including the brain, heart, liver, spleen, lung, kidney, adrenal gland, thymus, testis, epididymis, uterus, and ovary, were measured. All organs were weighed directly after dissection to avoid mechanical injury and stored in 10% neutral buffered formalin.

### Hematological Analyses

Blood samples were treated with EDTA-2K to perform hematological assessments. A HEMAVET 950 (Drew Scientific, NW, Miami Lakes, USA) automated hematology analyzer was used to measure the white blood cell (WBC) count, neutrophils (NE), lymphocytes (LY), monocytes (MO), red blood cell (RBC) count, hemoglobin (Hb), red blood cell distribution width (RDW), mean corpuscular volume (MCV), mean corpuscular hemoglobin (MCH), mean corpuscular hemoglobin concentration (MCHC), reticulocytes (RET), and platelets (PLT). Blood was treated with sodium citrate before prothrombin time (PT) was measured.

### Biochemical Analyses

Serum was obtained after centrifugation of blood at 3000 revolutions for 10 min and collected in 1.5 mL centrifuge tubes. In the serum, alanine aminotransferase (ALT), aspartate aminotransferase (AST), total protein (TP), albumin (ALB), total bilirubin (TBIL), alkaline phosphatase (ALP), blood urea nitrogen (BUN), creatinine (CRE), glucose (GLU), triglycerides (TG), total cholesterol (TC), sodium ions (Na^+^), potassium ions (K^+^), chloride ions (Cl^−^), ionized calcium (iCa), total calcium (TCa), and potential of hydrogen (pH) value were measured using a Beckman AU680 automatic biochemistry analyzer (CA, USA).

### Histopathological Examinations

The main organs (heart, liver, kidney, lung, spleen, stomach, prostate) from the control and 300 mg/kg bw groups at 7 and 13 weeks and after the recovery period were douched with running tap water for 4 h, dehydrated via a graded ethanol series, dewaxed in xylene, embedded in paraffin, cut into 5 µM sections using a microtome, and stained with hematoxylin and eosin (H&E). Moreover, both small intestine and colon were observed by H&E staining at the end of the week 7 treatment. The examination of additional organs such as the small intestine, colon, brain, adrenal gland, testis, ovary, and uterus was performed at the end of the week 13 administration. Pathological sections of tissues were examined under a Leica DM6000B light microscope and photomicrographs (Buffalo grove, IL, USA) obtained. When lesions were observed in the 300 mg/kg bw group, the diseased organs were also examined in the 100 mg/kg.bw group.

### Statistical Analyses

Data are presented as the mean ± standard deviation, and statistical analyses were performed using SPSS 17.0 software (Chicago, IL, USA). The variance in the data of all parameters was checked for homogeneity by using Bartlett’s procedure. When the data were homogeneous, one-way ANOVA was used. In the cases of heterogeneous data, the Kruskal–Wallis test was applied. When statistically significant differences were indicated, the LSD and Dunnett’s T3 multiple tests were employed for comparisons between control and treated groups. A nonparametric test was selected for the ranked data. Results were considered significant at p < 0.05.

## Electronic supplementary material

Below is the link to the electronic supplementary material.

Supplementary file1 (PDF 582 kb)

## Abbreviations

TA: Total alkaloids extract
Sch: Scholaricine
Epi: 19-Epischolaricine
Val: Vallesamine
Pic: Picrinine
Ech: Echitamine
H&E: Hematoxylin–Eosin
WBC: White blood cell count
NE: Neutrophils
LY: Lymphocytes
MO: Monocytes
RBC: Red blood cell count
Hb: Hemoglobin
RDW: Red blood cell distribution width
MCV: Mean corpuscular volume
MCH: Mean corpuscular hemoglobin
MCHC: Mean corpuscular hemoglobin concentration
PLT: Platelets
PT: Prothrombin time
RET: Reticulocytes
ALT: Alanine aminotransferase
AST: Aspartate aminotransferase
TP: Total protein
ALB: Albumin
TBIL: Total bilirubin
ALP: Alkaline phosphatase
BUN: Blood urea nitrogen
CRE: Creatinine
GLU: Glucose
TG: Triglycerides
TC: Total cholesterol
CK: Creatine kinase
K+: Potassium ions
Na^+^: Sodium ions
Cl^−^: Chloride ions
iCa: Ionized calcium
TCa: Total calcium
PH: Potential of hydrogen

## References

[1] Cosyns JP (2003). Drug Saf..

[2] Xue MZ, Zhang SD, Cai CC, Yu XJ, Shan L, Liu XF, Zhang WD, Li HL (2013). Evid.-Based Complement. Altern. Med..

[3] Han HY, Huh JI, Han SR, Kang MG, Yoon S, Han JS, Lee BS, Kim JA, Min BS (2018). Regul. Toxicol. Pharmacol..

[4] Langrand J, Regnault H, Cachet X, Bouzidi C, Villa AF, Serfaty L, Garnier R, Michel S (2014). Phytomedicine.

[5] P.T. Li, A.J.M. Leeuwenberg, D.J. Middleton. *Flora China*, vol 16 (Science Press, Beijing, 1995), p. 154

[6] C.G.O.Y.T.C. Medicine (Yunnan People's Press, Kunming, 1977)

[7] Cai XH, Shang JH, Feng T, Luo XD, Naturforsch Z (2010). Br. J. Chem. Sci..

[8] Chen YY, Yang J, Yang XW, Khan A, Liu L, Wang B, Zhao YL, Liu YP, Ding ZT, Luo XD (2016). Tetrahedron Lett..

[9] Yang XW, Song CW, Zhang Y, Khan A, Jiang LP, Chen YB, Liu YP, Luo XD (2015). Tetrahedron Lett..

[10] Yang XW, Qin XJ, Zhao YL, Lunga PK, Li XN, Jiang SZ, Cheng GG, Liu YP, Luo XD (2014). Tetrahedron Lett..

[11] Yang XW, Luo XD, Lunga PK, Zhao YL, Qin XJ, Chen YY, Liu L, Li XN, Liu YP (2015). Tetrahedron.

[12] Qin XJ, Zhao YL, Lunga PK, Yang XW, Song CW, Cheng GG, Liu L, Chen YY, Liu YP, Luo XD (2015). Tetrahedron.

[13] Feng T, Cai XH, Zhao PJ, Du ZZ, Li WQ, Luo XD (2009). Planta Med..

[14] Yang XW, Yang CP, Jiang LP, Qin XJ, Liu YP, Shen QS, Chen YB, Luo XD (2014). Org. Lett..

[15] Pan ZQ, Qin XJ, Liu YP, Wu T, Luo XD, Xia CF (2016). Org. Lett..

[16] Cai XH, Du ZZ, Luo XD (2007). Org. Lett..

[17] Cai XH, Tan QG, Liu YP, Feng T, Du ZZ, Li WQ, Luo XD (2008). Org. Lett..

[18] Qin XJ, Zhao YL, Song CW, Wang B, Chen YY, Liu L, Li Q, Li D, Liu YP, Luo XD (2015). Nat. Prod. Bioprospect..

[19] Zhang ZY, Luo XD, Li S (2014). J. Med. Plants Res..

[20] Zhou H, He HP, Luo XD, Wang YH, Yang XW, Di YT, Hao XJ (2005). Helv. Chim. Acta.

[21] Feng T, Cai XH, Du ZZ, Luo XD (2008). Helv. Chim. Acta.

[22] Liu L, Chen YY, Qin XJ, Wang B, Jin Q, Liu YP, Luo XD (2015). Fitoterapia.

[23] Xu Y, Feng T, Cai XH, Luo XD (2009). Chin. J. Nat. Med..

[24] Du GS, Cai XH, Shang JH, Luo XD (2007). Chin. J. Nat. Med..

[25] Cai XH, Liu YP, Feng T, Luo XD (2008). Chin. J. Nat. Med..

[26] Du GS, Shang JH, Cai XH, Luo XD (2007). Acta Bot. Yunnanica.

[27] Cao J, Shen HM, Wang Q, Qian Y, Guo HC, Li K, Qiao X, Guo DA, Luo XD, Ye M (2015). J Chromatogr B Analyt. Technol. Biomed. Life Sci..

[28] Zhao YL, Shang JH, Liu YP, Liu L, Cao J, Qian Y, Khan A, Wang HS, Ye M, Luo XD (2017). Phytomedicine.

[29] Shang JH, Cai XH, Feng T, Zhao YL, Wang JK, Zhang LY, Yan M, Luo XD (2010). J. Ethnopharmacol..

[30] Shang JH, Cai XH, Zhao YL, Feng T, Luo XD (2010). J. Ethnopharmacol..

[31] Zhao YL, Shang JH, Pu SB, Wang HS, Wang B, Liu L, Liu YP, Mei SH, Luo XD (2016). J. Ethnopharmacol..

[32] Zhao YL, Cao J, Shang JH, Liu YP, Khan A, Wang HS, Qian Y, Liu L, Ye M, Luo XD (2017). Phytomedicine.

[33] Zhao YL, Yang ZF, Shang JH, Huang WY, Wang B, Wei X, Khan A, Yuan ZW, Liu YP, Wang YF, Wang XH, Luo XD (2018). J. Ethnopharmacol..

[34] Hou YY, Cao XL, Dong LY, Wang LQ, Cheng BF, Shi Q, Luo XD, Bai G (2012). J. Chromatogr. A.

[35] Hou YY, Cao XL, Wang LQ, Cheng BF, Dong LY, Luo XD, Bai G, Gao WY (2012). J. Chromatogr. B.

[36] Baliga MS, Jagetia GC, Ulloor JN, Baliga MP, Venkatesh P, Reddy R, Rao KV, Baliga BS, Devi S, Raju SK, Veeresh V, Reddy TK, Bairy KL (2004). Toxicol. Lett..

[37] Jagetia GC, Baliga MS, Venkatesh P, Ulloor JN, Mantena SK, Genebriera J, Mathuram V (2005). J. Pharm. Pharmacol..

[38] El Hilaly J, Israili ZH, Badiaa L (2004). J. Ethnopharmacol..

[39] JM. Lee, MA Lee, HN Do, YI Song, RJ Bae, HY Lee, SH Park, JS Kang, JK Kang, Lab. Anim. Res. **28**, 115–121 (2012) 10.5625/lar.2012.28.2.115 PMC3389835 22787485

[40] Sireeratawong S, Jaijoy K, Khonsung P, Lertprasertsuk N, Ingkaninan K (2016). BMC Complement. Altern. Med..

[41] Jin SE, Seo C-S, Lee M-Y, Shin H-K, Yang MJ, Ha H (2018). J. Ethnopharmacol..

[42] Nanna U, Jaijoy K, Lertprasertsuke N, Soonthornchareonnon N, Sireeratawong S (2015). J. Med. Assoc. Thai..

[43] Qin GQ, Tang S, Li SB, Lu HL, Wang YW, Zhao P, Li B, Zhang JH, Peng L (2016). Environ. Toxicol..

[44] OECD, The OECD guideline for testing of chemical: chronic toxicity studies. In No. 452, development, O. f. E. C.-o. a., Ed. Paris (1981)

[45] Huang JN, Chen XJ, Lin S (2012). Progr. Vet. Med..

