# Supplementary material for: Acute and Chronic Toxicity of Indole Alkaloids from Leaves of Alstonia scholaris (L.) R. Br. in Mice and Rats
Source: Nat Prod Bioprospect. 2020 Mar 31;10(2):77–88. doi: 10.1007/s13659-020-00237-1 (PMC7176796; doi:10.1007/s13659-020-00237-1)
Supplement: Supplementary file 1 — Supplementary file1 (PDF 582 kb) [file 13659_2020_237_MOESM1_ESM.pdf]

Supporting Information for

Acute and chronic toxicity of indole alkaloids from *Alstonia scholaris* (L.) R. Br. in mice and rats

Yun-Li Zhao [a,c,1], Min Su [b,1], Jian-Hua Shang [b,c,1], Xia Wang [b], Guy Sedar Singor Njateng [d], Guang-Lei Bao [b], Jia Ma [b], Qing-Di Sun [e], Fang Yuan [b], Jing-Kun Wang [b,*], Xiao-Dong Luo [a,c,*]

[a] *State Key Laboratory of Phytochemistry and Plant Resources in West China, Kunming Institute of Botany, Chinese Academy of Sciences, Kunming 650201, P. R. China*

[b] *Yunnan Institute of Medical Material, Kunming 650111, P. R. China*

[c] *Key Laboratory of Medicinal Chemistry for Natural Resource, Ministry of Education and Yunnan Province, School of Chemical Science and Technology, Yunnan University, Kunming 650091, People's Republic of China*

[d] *Laboratory of Microbiology and Antimicrobial Substances, Faculty of Science, University of Dschang, P.O. Box 67 Dschang, Cameroon*

[e] *Jiangsu Nhwa Pharmaceutical Co., Ltd, Xuzhou, 221009, P. R. China*

---

* Corresponding author. Tel.: +86 871 65223177; fax: +86 871 65220227.

E-mail address: xdluo@mail.kib.ac.cn (X.-D. Luo); wjkyimm@163.com (J.-K. Wang)

[1]These authors contributed equally.

Supplementary Text

S1. Alkaloids quantitative analysis 3

S1.1. Sample preparation 3

S1.2. HPLC/UV quantitative analysis 3

Table S1 Mean food intake of rats after treatment with TA 4

Table S2 Mean water consumption of rats after treatment with TA 5

Table S3 The body weight of female rats after treatment with TA 6

Table S4 The body weight of male rats after treatment with TA 7

Table S5 Hematological examination of rats treated with TA for 7 weeks 8

Table S6 Hematological examination of rats treated with TA for 13 weeks 9

Table S7 Hematological examination of rats after the 4-week recovery period 10

Table S8 Serum chemistry values of rats treated with TA for 7 weeks 11

Table S9 Serum chemistry values of rats treated with TA for 13 weeks 13

Table S10 Serum chemistry values of rats after the 4-week recovery period 15

Table S11 Organ weights and coefficients of rats treated with TA for 7 weeks 17

Table S12 Organ weights and coefficients of rats treated with TA for 13 weeks 19

Table S13 Organ weights and coefficients of rats after the 4-week recovery period 21

Fig. S1. Representative sections of rats in respect of 7 weeks treatment demonstrating histopathology 23

Fig. S2. Representative sections of rats in respect of 13 weeks treatment demonstrating histopathology 25

Fig. S3. Representative sections of rats in the recovery period demonstrating histopathology 27

References 28

S1. Alkaloids quantitative analysis

S1.1. Sample preparation

The sample of total alkaloids was pulverized into fine powder. Then 1.0 mg of the total alkaloids was accurately weighed and extracted with 1.0 mL of 50% methanol in ultrasonic water bath for 30 min at 25 °C. The sample solution was filtered through a 0.22 μm membrane before use. A 10 μL-aliquot was injected for UHPLC/UV quantitative analysis.

S1.2. HPLC/UV quantitative analysis

The quantitative analysis was performed on an a Agilent 1290 series HPLC system (Agilent Technologies, Germany) comprised a quaternary pump, an auto-sampler, a column temperature controller and a PDA detector. All samples were separated on an ACQUITY UPLC® CSH column (2.1 × 100 mm, 1.7 μm) equipped with a Waters VanGuard pre-column (5 mm × 2.1 mm I.D., 1.8 μm). The mobile phase consisted of acetonitrile (A) and water containing 0.5% trifluoroacetic acid (v/v, B) at the ratio of 18 : 82. The following linear elution gradient was used: 0-10 min, 4-16% A; 10-15 min, 16-20% A; 15-18 min, 20% A; 18-20min, 20-95% A. The flow rate was 0.20 mL/min. The column temperature was maintained at 45 °C. The sample tray temperature was maintained at 25 °C. The detection wavelength was set from 190 to 600 nm, and the samples were detected at 285 nm in accordance with the maximum absorption of the analytes.

Table S1 Mean food intake of rats after treatment with TA

Rats	Weeks	Control	50 mg/kg.bw	100 mg/kg.bw	300 mg/kg.bw
Female	1	96.85 ± 8.68	100.04 ± 19.22	90.38 ± 4.02	117.64 ± 43.94
	2	89.43 ± 3.55	89.59 ± 14.93	87.92 ± 17.29	103.80 ± 28.15
	3	88.49 ± 9.17	89.93 ± 12.65	85.32 ± 6.15	98.55 ± 26.57
	4	79.64 ± 6.57	88.37 ± 12.21	85.37 ± 12.68	102.58 ± 35.90
	5	101.82 ± 37.45	77.36 ± 5.65	82.62 ± 12.90	104.98 ± 51.85
	6	101.02 ± 44.82	80.61 ± 6.35	80.41 ± 8.93	100.49 ± 36.08
	7	90.19 ± 26.48	87.32 ± 29.09	89.47 ± 21.14	86.15 ± 18.60
	8	104.29 ± 33.91	84.24 ± 34.93	71.34 ± 17.97	70.22 ± 8.82
	9	70.47 ± 10.92	82.47 ± 29.79	77.43 ± 18.84	69.21 ± 7.26
	10	82.47 ± 20.40	74.94 ± 19.74	87.02 ± 15.92	91.27 ± 31.31
	11	68.91 ± 10.53	69.26 ± 15.89	72.68 ± 11.72	67.50 ± 4.36
	12	59.43 ± 8.39	61.43 ± 2.82	64.95 ± 6.75	70.96 ± 14.97
	13	69.19 ± 10.58	62.15 ± 2.13	65.49 ± 12.73	69.06 ± 9.28
	14	61.54 ± 4.63	57.35 ± 2.51	47.10 ± 0.63	45.84 ± 5.83
	15	57.05 ± 3.99	56.04 ± 3.74	61.02 ± 6.14	78.90 ± 29.19
	16	60.71 ± 6.84	59.54 ± 2.29	54.33 ± 33.81	42.33 ± 20.97
	17	63.05 ± 7.52	63.52 ± 4.47	55.70 ± 0.72	79.82 ± 26.28
Male	1	86.34 ± 8.14	89.29 ± 3.87	91.97 ± 3.08	83.89 ± 15.00
	2	84.08 ± 5.19	84.51 ± 4.49	87.55 ± 3.64	81.65 ± 7.49
	3	72.58 ± 2.99	80.64 ± 2.54$^{*\triangledown}$	79.44 ± 1.80$^{*\triangledown}$	72.45 ± 7.75
	4	68.24 ± 6.59	70.60 ± 1.12	73.97 ± 2.77	65.99 ± 6.05
	5	63.48 ± 6.29	68.34 ± 1.45	68.69 ± 0.98	66.89 ± 7.31
	6	61.30 ± 6.79	65.50 ± 1.88	67.17 ± 1.51	64.59 ± 7.02
	7	63.88 ± 4.15	61.60 ± 1.39	59.95 ± 5.09	58.83 ± 5.43
	8	58.91 ± 5.72	51.56 ± 5.24$^{*\blacktriangle\triangledown\triangledown}$	57.37 ± 1.27	59.63 ± 0.95
	9	58.78 ± 2.33	58.72 ± 2.74	58.62 ± 1.46	60.30 ± 1.28
	10	56.07 ± 1.94	55.81 ± 0.78	53.28 ± 4.69	57.10 ± 0.89
	11	54.07 ± 3.77	53.78 ± 2.16	54.43 ± 1.79	57.23 ± 2.30
	12	50.98 ± 3.49	51.32 ± 1.43	51.95 ± 0.81	53.47 ± 1.52
	13	51.54 ± 3.02	50.55 ± 1.02	50.19 ± 0.68	49.85 ± 3.45
	14	48.87 ± 1.76	43.39 ± 2.12	46.90 ± 1.23	48.55 ± 1.69
	15	45.20 ± 3.39	47.65 ± 2.35	47.34 ± 2.92	50.30 ± 1.05
	16	49.39 ± 2.23	45.19 ± 0.19	45.17 ± 5.09	48.40 ± 1.47
	17	49.16 ± 0.57	46.71 ± 0.17	50.01 ± 3.52	51.06 ± 1.10

Results were expressed as means ± SD. n: the number of rats. n = 20 at 7 week; n = 15 at 13 week; n = 5 at 17 week for single-sex.

* $p < 0.05$ was compared with the control group.

$^{\blacktriangle}$ $p < 0.05$ was compared with the 100 mg/kg.bw group.

$^{\triangledown/\triangledown\triangledown}$ $p < 0.05/0.01$ was compared with the 300 mg/kg.bw group.

Unit: g/kg/d.

Table S2 Mean water consumption of rats after treatment with TA

Rats	Weeks	Control	50 mg/kg.bw	100 mg/kg.bw	300 mg/kg.bw
Female	1	136.30 ± 13.45	120.59 ± 5.23▲▲▽	145.44 ± 19.54	138.96 ± 9.58
	2	155.16 ± 11.41	127.32 ± 23.07	164.24 ± 29.75	148.46 ± 6.92
	3	139.25 ± 16.02	122.38 ± 6.51▲▲▽	155.93 ± 20.23	143.86 ± 14.41
	4	136.69 ± 11.89	132.17 ± 19.23	156.77 ± 29.18	131.58 ± 17.54
	5	132.33 ± 10.11	109.72 ± 7.29*▲▲	142.01 ± 28.92	108.36 ± 11.98*▲▲
	6	139.21 ± 10.53	112.88 ± 10.98*▲▽	143.53 ± 29.34	127.51 ± 7.61
	7	134.51 ± 11.96	99.10 ± 10.70*▲	142.37 ± 37.53	104.78 ± 13.83*
	8	115.00 ± 15.11	91.54 ± 8.66*▲	115.29 ± 18.19	91.14 ± 12.92*▲
	9	109.63 ± 10.59	109.22 ± 17.78	120.40 ± 18.40	110.88 ± 9.98
	10	126.21 ± 29.99	110.46 ± 32.15	142.25 ± 53.76	117.71 ± 38.73
	11	106.71 ± 13.78	84.86 ± 16.00	108.33 ± 29.37	91.04 ± 14.70
	12	111.15 ± 15.37	97.09 ± 27.37	109.66 ± 24.22	100.77 ± 12.32
	13	109.10 ± 20.04	92.76 ± 11.42	111.85 ± 25.18	86.09 ± 18.77
	14	98.20 ± 16.91	92.66 ± 10.65	84.50 ± 20.89	89.99 ± 12.81
	15	112.08 ± 22.05	112.40 ± 17.18	102.36 ± 15.26	103.83 ± 10.75
	16	87.05 ± 17.05	97.96 ± 32.29	93.60 ± 35.29	81.40 ± 7.10
	17	82.64 ± 19.17	82.45 ± 2.34	83.98 ± 19.08	87.45 ± 1.38
Male	1	143.51 ± 6.54	136.17 ± 9.03	145.42 ± 17.90	167.84 ± 41.28
	2	145.43 ± 15.05	138.73 ± 21.90	144.78 ± 13.35	152.59 ± 14.53
	3	134.83 ± 7.12	122.74 ± 10.05▲▲▽	144.37 ± 8.66	138.47 ± 15.00
	4	142.20 ± 9.91	128.69 ± 18.37	134.51 ± 6.81	140.33 ± 18.47
	5	117.44 ± 13.98	106.00 ± 10.09	120.79 ± 16.25	119.99 ± 17.95
	6	109.08 ± 20.62	107.43 ± 19.00	115.09 ± 6.42	117.46 ± 16.15
	7	112.43 ± 13.03	101.26 ± 10.30	122.52 ± 19.29	112.07 ± 16.72
	8	99.94 ± 10.65	82.94 ± 9.87	96.69 ± 14.70	96.57 ± 15.17
	9	101.63 ± 13.59	84.85 ± 8.16	87.85 ± 9.02	91.45 ± 20.35
	10	103.14 ± 15.12	83.38 ± 10.80	83.63 ± 5.27	105.11 ± 25.25
	11	94..95 ± 14.35	70.76 ± 9.47**▽	75.09 ± 5.85**	87.82 ± 10.94
	12	89.57 ± 17.99	72.85 ± 5.10	77.96 ± 10.58	87.18 ± 15.01
	13	90.86 ± 17.41	66.96 ± 11.55	70.89 ± 10.91	86.17 ± 20.21
	14	71.61 ± 16.28	72.11 ± 7.85	77.91 ± 9.15	73.02 ± 8.31
	15	75.11 ± 8.23	78.10 ± 13.27	82.68 ± 1.32	103.31 ± 26.98
	16	67.02 ± 21.64	65.76 ± 13.42	65.58 ± 10.00	62.10 ± 18.46
	17	61.71 ± 16.61	64.60 ± 16.21	67.11 ± 0.84	86.38 ± 4.17

Results were expressed as means ± SD. n: the number of rats. n = 20 at 7 week; n = 15 at 13 week; n = 5 at 17 week for single-sex.

*/** $p < 0.05/0.01$ was compared with the control group.

▲/▲▲ $p < 0.05/0.01$ was compared with the 100 mg/kg.bw group.

▽ $p < 0.05$ was compared with the 300 mg/kg.bw group.

Unit: mL/kg/d.

Table S3 The body weight of female rats after treatment with TA

Weeks	Control	50 mg/kg.bw	100 mg/kg.bw	300 mg/kg.bw
0	140.04 ± 6.91	140.04 ± 8.26	140.02 ± 7.12	140.75 ± 7.80
0.5	154.28 ± 8.77	152.54 ± 8.14	151.67 ± 9.40	150.47 ± 9.76
1	168.56 ± 9.62	167.13 ± 8.78	166.13 ± 9.01	164.68 ± 10.17
1.5	178.98 ± 11.68	177.66 ± 10.52	173.90 ± 11.89	173.37 ± 11.80
2	189.80 ± 12.38	189.44 ± 11.23	185.52 ± 9.49	184.38 ± 12.54
2.5	199.80 ± 14.19	197.61 ± 12.50	195.33 ± 9.69	194.92 ± 12.59
3	209.94 ± 15.72	210.50 ± 13.77	205.16 ± 10.78	204.57 ± 12.91
3.5	216.26 ± 17.48	215.96 ± 15.21	214.25 ± 11.24	214.28 ± 14.52
4	227.42 ± 18.93	225.10 ± 16.22	223.58 ± 11.34	222.55 ± 16.19
4.5	234.12 ± 19.10	230.72 ± 16.68	230.12 ± 12.92	228.55 ± 16.82
5	241.44 ± 19.55	241.08 ± 14.89	237.91 ± 13.54	234.14 ± 18.30
5.5	250.25 ± 21.43	249.06 ± 17.77	245.04 ± 12.83	239.51 ± 17.71
6	254.90 ± 20.89	253.54 ± 17.12	249.30 ± 11.57	243.55 ± 18.11
6.5	259.70 ± 22.39	259.28 ± 17.48	255.58 ± 13.08	239.51 ± 17.71$^{**\Delta\Delta▲▲}$
7	261.14 ± 22.26	260.54 ± 16.30	256.51 ± 11.61	248.88 ± 21.31
7.5	267.52 ± 24.23	260.71 ± 13.58	260.58 ± 13.49	257.20 ± 20.67
8	273.74 ± 24.63	266.00 ± 13.42	266.37 ± 14.19	261.56 ± 19.84
8.5	277.25 ± 25.93	275.39 ± 15.65	269.47 ± 16.06	265.25 ± 20.15
9	281.21 ± 26.70	276.76 ± 16.51	273.05 ± 15.83	266.51 ± 19.75
9.5	284.37 ± 26.05	281.61 ± 18.00	275.65 ± 17.05	268.72 ± 21.00
10	287.43 ± 26.40	285.99 ± 19.01	278.40 ± 17.52	272.17 ± 20.41
10.5	289.47 ± 25.74	287.69 ± 17.45	281.97 ± 16.99	276.23 ± 19.06
11	292.69 ± 26.67	291.14 ± 17.48	284.61 ± 18.13	276.76 ± 20.24
11.5	295.45 ± 29.16	293.05 ± 17.90	287.12 ± 19.15	276.34 ± 23.21
12	301.00 ± 28.76	296.31 ± 17.56	292.13 ± 19.08	280.61 ± 24.34
12.5	302.18 ± 30.39	298.77 ± 19.79	294.51 ± 20.14	283.10 ± 26.91
13	293.85 ± 29.95	292.15 ± 18.96	287.66 ± 18.17	276.49 ± 23.43
13.5	311.58 ± 38.54	299.10 ± 19.41	296.50 ± 10.01	286.08 ± 21.97
14	315.56 ± 41.07	300.58 ± 19.85	300.82 ± 11.23	291.58 ± 21.17
14.5	319.62 ± 39.18	303.82 ± 17.88	303.98 ± 13.26	292.54 ± 16.04
15	321.12 ± 41.36	306.74 ± 17.69	310.14 ± 16.57	295.90 ± 15.07
15.5	330.04 ± 44.76	308.24 ± 15.66	311.42 ± 13.84	296.76 ± 19.98
16	327.74 ± 44.22	309.06 ± 18.24	311.96 ± 15.78	301.88 ± 17.57
16.5	332.76 ± 44.92	311.86 ± 15.97	314.74 ± 16.74	305.78 ± 14.57
17	322.72 ± 43.19	302.92 ± 15.80	304.90 ± 17.59	298.74 ± 19.04

Results were expressed as means ± SD. n: the number of rats. n = 20 at 7 week; n = 15 at 13 week; n = 5 at 17 week for single-sex.

** $p < 0.01$ was compared with the control group.

$^{\Delta\Delta}$ $p < 0.01$ was compared with the 50 mg/kg.bw group.

▲▲ $p < 0.01$ was compared with the 100 mg/kg.bw group.

Unit: g.

Table S4 The body weight of male rats after treatment with TA

Weeks	Control	50 mg/kg.bw	100 mg/kg.bw	300 mg/kg.bw
0	170.90 ± 9.17	171.12 ± 10.27	170.82 ± 8.04	170.87 ± 10.11
0.5	192.42 ± 9.72	194.28 ± 9.64	192.88 ± 7.70	191.67 ± 8.83
1	219.54 ± 15.78	220.22 ± 13.79	216.87 ± 9.72	218.48 ± 8.88
1.5	240.22 ± 16.86	240.92 ± 14.49	236.59 ± 12.57	239.36 ± 8.77
2	266.06 ± 17.72	262.62 ± 17.27	258.23 ± 17.09	262.09 ± 9.93
2.5	284.00 ± 22.79	278.26 ± 19.60	275.98 ± 19.27	276.90 ± 11.86
3	302.58 ± 30.24	295.18 ± 22.60	294.13 ± 23.04	292.69 ± 13.81
3.5	319.60 ± 31.69	308.56 ± 25.42	310.26 ± 24.83	305.76 ± 15.27
4	336.99 ± 32.19	324.79 ± 29.38	324.14 ± 26.02	318.70 ± 17.42
4.5	350.95 ± 34.83	334.50 ± 30.98	335.52 ± 28.46	329.44 ± 20.21
5	366.01 ± 36.78	349.00 ± 32.39	348.66 ± 29.41	343.62 ± 21.93
5.5	380.01 ± 38.12	362.06 ± 33.96	361.32 ± 30.36	356.44 ± 24.06
6	392.62 ± 39.34	372.31 ± 35.05	373.26 ± 28.69	368.30 ± 25.78
6.5	405.02 ± 38.54	384.26 ± 35.16	385.93 ± 29.60	378.10 ± 26.86
7	413.77 ± 41.55	390.66 ± 37.45	394.04 ± 33.54	383.93 ± 28.58
7.5	428.19 ± 44.96	406.31 ± 36.06	410.87 ± 34.51	394.93 ± 26.38
8	442.02 ± 44.27	419.91 ± 35.70	424.67 ± 33.65	406.53 ± 27.43
8.5	453.11 ± 45.99	430.71 ± 36.27	434.63 ± 34.12	416.03 ± 27.91
9	464.25 ± 47.48	439.33 ± 37.36	445.39 ± 32.45	424.73 ± 27.96**
9.5	471.28 ± 47.64	446.97 ± 37.96	453.13 ± 31.44	433.86 ± 29.25
10	480.67 ± 48.00	453.49 ± 38.08	459.27 ± 32.33	441.27 ± 29.92
10.5	488.15 ± 47.44	462.39 ± 38.13	468.75 ± 34.73	450.65 ± 32.97
11	496.75 ± 48.98	470.04 ± 39.61	477.01 ± 34.32	452.37 ± 48.08
11.5	501.81 ± 48.70	473.25 ± 37.16	483.94 ± 33.27	465.87 ± 35.95
12	510.91 ± 48.89	480.01 ± 37.85*	488.19 ± 33.02	470.71 ± 37.64**
12.5	515.74 ± 49.72	483.63 ± 37.87*	492.09 ± 33.09	472.09 ± 38.43**
13	505.69 ± 50.86	474.05 ± 33.69*	484.21 ± 33.09	465.16 ± 40.56**
0.5	535.62 ± 75.67	468.64 ± 35.87	505.16 ± 40.89	509.36 ± 37.57
1	545.58 ± 79.25	479.92 ± 36.84	517.86 ± 42.28	519.78 ± 38.50
1.5	551.82 ± 78.93	483.02 ± 36.06	522.42 ± 39.97	525.24 ± 36.96
2	548.28 ± 83.08	487.62 ± 32.74	525.62 ± 39.25	518.64 ± 38.68
2.5	565.94 ± 83.61	493.26 ± 33.99	531.92 ± 38.73	534.48 ± 37.82
3	574.84 ± 85.07	498.94 ± 34.49	535.32 ± 40.07	539.74 ± 41.41
3.5	578.54 ± 89.57	501.82 ± 32.04	541.40 ± 41.25	546.98 ± 42.39
4	566.36 ± 83.17	492.38 ± 32.03	524.84 ± 39.70	529.46 ± 38.42

Results were expressed as means ± SD. n, the number of rats. n = 20 at 7 week; n = 15 at 13 week; n = 5 at 17 week for single-sex.

$^{*/**}$ $p < 0.05/0.01$ was compared with the control group.

Unit: g.

Table S5 Hematological examination of rats treated with TA for 7 weeks

Rats	Indexes	Control	50 mg/kg.bw	100 mg/kg.bw	300 mg/kg.bw
Female (n = 5)	WBC ($\times 10^{9}$/L)	6.22 ± 1.48	6.12 ± 1.36	6.20 ± 1.36	6.49 ± 2.48
	NE%	34.86 ± 4.14	33.75 ± 1.93	29.63 ± 3.48	33.35 ± 2.97
	LY%	58.70 ± 6.21	59.12 ± 5.15	65.66 ± 3.77	61.63 ± 2.34
	MO%	4.52 ± 5.09	5.50 ± 5.30	2.72 ± 0.34	2.78 ± 0.43
	RBC ($\times 10^{12}$/L)	6.15 ± 0.64	6.58 ± 0.34	6.03 ± 0.71	6.42 ± 0.30
	Hb (g/L)	124.80 ± 10.06	132.00 ± 5.10	126.00 ± 7.07	133.20 ± 3.03
	RDW (%)	17.42 ± 1.22	16.56 ± 0.61	18.00 ± 1.46	16.74 ± 0.54
	MCV (fL)	45.86 ± 2.71	44.58 ± 2.07	45.82 ± 3.18	46.08 ± 1.59
	MCH (pg)	20.28 ± 0.97	20.04 ± 0.78	21.06 ± 1.52	20.76 ± 0.68
	MCHC (g/L)	446.80 ± 14.96	448.80 ± 8.04	459.60 ± 3.13	453.80 ± 2.95
	PLT ($\times 10^{9}$/L)	914.80 ± 192.32	853.20 ± 30.74	859.20 ± 75.96	915.20 ± 133.87
	PT (Sec)	13.46 ± 0.48	12.98 ± 0.27$^{*\blacktriangle\triangledown}$	14.62 ± 1.30*	14.40 ± 0.92*
	RET (%)	1.10 ± 0.20	1.44 ± 0.57	1.68 ± 0.44	1.42 ± 0.54
Male (n = 5)	WBC ($\times 10^{9}$/L)	6.59 ± 1.30	6.76 ± 0.74	7.41 ± 1.56	9.43 ± 1.56$^{**\Delta\Delta\blacktriangle}$
	NE%	28.74 ± 1.42	30.72 ± 2.09	30.91 ± 2.11	30.85 ± 1.62
	LY%	64.95 ± 2.99	62.52 ± 2.13	63.46 ± 1.86	63.45 ± 2.02
	MO%	3.70 ± 0.95	4.18 ± 0.63	3.13 ± 0.59	3.37 ± 0.44
	RBC ($\times 10^{12}$/L)	7.12 ± 0.40	7.39 ± 0.51	7.20 ± 0.46	7.10 ± 0.34
	Hb (g/L)	135.60 ± 2.97	140.40 ± 8.53	138.00 ± 6.16	138.40 ± 2.97
	RDW (%)	17.20 ± 0.67	17.92 ± 0.68	17.34 ± 0.27	17.52 ± 1.09
	MCV (fL)	41.40 ± 1.34	42.08 ± 0.61	41.70 ± 1.66	42.98 ± 1.56
	MCH (pg)	19.10 ± 0.71	18.98 ± 0.70	19.14 ± 0.78	19.48 ± 0.59
	MCHC (g/L)	463.20 ± 3.03	456.00 ± 12.41	459.40 ± 4.50	454.40 ± 5.37
	PLT ($\times 10^{9}$/L)	931.20 ± 77.36	864.40 ± 40.38	903.20 ± 139.17	843.20 ± 81.74
	PT (Sec)	14.06 ± 1.49	14.08 ± 1.81	13.64 ± 0.27	13.56 ± 0.38
	RET (%)	2.20 ± 0.69	1.58 ± 0.58	1.80 ± 0.87	1.42 ± 0.74

Results were expressed as means ± SD.

$^{*/**}$ $p < 0.05/0.01$ was compared with the control group.

$^{\Delta\Delta}$ $p < 0.01$ was compared with the 50 mg/kg.bw group.

$^{\blacktriangle}$$p < 0.05$ was compared with the 100 mg/kg.bw group.

$^{\triangledown}$ $p < 0.05$ was compared with the 300 mg/kg.bw group.

Table S6 Hematological examination of rats treated with TA for 13 weeks

Rats	Indexes	Control	50 mg/kg.bw	100 mg/kg.bw	300 mg/kg.bw
Female (n = 10)	WBC (×10^9/L)	5.24 ± 1.25	5.49 ± 1.04	6.03 ± 1.78	7.15 ± 1.14$^{**\Delta\Delta}$
	NE%	30.34 ± 1.55	30.08 ± 1.67	29.40 ± 3.59	31.01 ± 3.34
	LY%	63.88 ± 1.14	62.82 ± 1.69	63.63 ± 2.93	62.68 ± 2.72
	MO%	3.45 ± 0.82	4.32 ± 0.57$^{*\nabla}$	4.32 ± 0.93$^{*\nabla}$	3.46 ± 0.60
	RBC (×10^{12}/L)	6.49 ± 0.29	6.14 ± 0.52	6.29 ± 0.48	6.24 ± 0.75
	Hb (g/L)	130.00 ± 5.33	127.00 ± 10.30	125.40 ± 7.37	157.10 ± 62.37
	RDW (%)	18.33 ± 0.76	18.32 ± 0.92	18.06 ± 0.36	17.18 ± 1.50$^{*\Delta}$
	MCV (fL)	44.63 ± 1.32	45.17 ± 1.25	45.22 ± 1.87	44.00 ± 9.10
	MCH (pg)	20.01 ± 0.70	19.63 ± 3.07	20.23 ± 0.90	20.55 ± 1.44
	MCHC (g/L)	446.30 ± 8.15	432.30 ± 64.83	420.90 ± 84.82	377.90 ± 112.88
	PLT (×10^9/L)	856.40 ± 113.82	811.50 ± 64.16	799.60 ± 118.06	791.80 ± 81.55
	PT (Sec)	15.00 ± 0.76	14.84 ± 0.37	14.71 ± 1.16	15.30 ± 1.44
	RET (%)	1.63 ± 0.33	1.56 ± 0.62	1.69 ± 0.57	1.49 ± 0.42
Male (n = 10)	WBC (×10^9/L)	9.13 ± 2.72	7.48 ± 1.01	6.56 ± 2.27$^{*\nabla}$	9.00 ± 2.07
	NE%	31.65 ± 2.67	30.42 ± 1.16	31.02 ± 1.18	31.48 ± 1.21
	LY%	61.66 ± 2.58	62.54 ± 1.62	62.24 ± 1.08	62.31 ± 1.01
	MO%	3.98 ± 0.61	4.22 ± 0.55	3.95 ± 0.33	3.73 ± 0.65
	RBC (×10^{12}/L)	7.37 ± 0.65	7.58 ± 0.78	7.56 ± 0.70	7.00 ± 0.82
	Hb (g/L)	137.40 ± 5.34	137.20 ± 5.43	155.20 ± 50.45	131.60 ± 9.46
	RDW (%)	18.07 ± 0.55	18.02 ± 0.77	17.58 ± 0.89	18.08 ± 0.92
	MCV (fL)	41.85 ± 1.74	42.33 ± 2.44	41.04 ± 2.14	42.90 ± 1.69
	MCH (pg)	18.66 ± 1.11	18.42 ± 1.35	18.22 ± 0.90	18.94 ± 1.03
	MCHC (g/L)	446.90 ± 10.78	435.80 ± 11.56	418.70 ± 80.74	443.80 ± 9.50
	PLT (×10^9/L)	878.80 ± 63.10	872.80 ± 52.13	818.80 ± 40.74	868.20 ± 96.41
	PT (Sec)	15.10 ± 0.59	15.00 ± 0.65	14.56 ± 0.54	14.84 ± 0.66
	RET (%)	1.40 ± 0.26	1.50 ± 0.44	1.61 ± 0.33	1.62 ± 0.33

Results were expressed as means ± SD.

$^{*/**}$ $p < 0.05/0.01$ was compared with the control group.

$^{\Delta/\Delta\Delta}$ $p < 0.05/0.01$ was compared with the 50 mg/kg.bw group.

$^{\nabla}$ $p < 0.05$ was compared with the 300 mg/kg.bw group.

Table S7 Hematological examination of rats after the 4-week recovery period

Rats	Indexes	Control	50 mg/kg.bw	100 mg/kg.bw	300 mg/kg.bw
Female (n = 5)	WBC ($\times 10^9$/L)	5.18 ± 0.91	5.50 ± 0.87	6.38 ± 1.79	5.22 ± 1.05
	NE%	31.82 ± 0.94	32.19 ± 1.68	31.00 ± 1.18	31.22 ± 0.96
	LY%	62.42 ± 0.76	61.63 ± 1.70	61.42 ± 1.65	61.98 ± 1.09
	MO%	3.77 ± 0.44	4.27 ± 0.87	4.87 ± 1.18	4.40 ± 0.71
	RBC ($\times 10^{12}$/L)	6.17 ± 0.30	6.12 ± 0.34	6.17 ± 0.29	6.78 ± 0.50$^{*\Delta▲}$
	Hb (g/L)	124.80 ± 3.63	127.20 ± 3.35	125.20 ± 4.15	134.80 ± 3.03$^{*\Delta▲}$
	RDW (%)	17.68 ± 0.90	17.92 ± 0.91	18.12 ± 0.70	17.68 ± 0.55
	MCV (fL)	45.14 ± 1.32	46.14 ± 1.11	45.64 ± 0.97	44.74 ± 2.08
	MCH (pg)	20.24 ± 0.63	20.88 ± 0.57	20.34 ± 0.59	19.96 ± 0.97
	MCHC (g/L)	448.60 ± 2.70	449.00 ± 6.36	443.80 ± 3.56	443.80 ± 3.42
	PLT ($\times 10^9$/L)	808.00 ± 64.06	850.40 ± 73.86	787.60 ± 83.41	858.80 ± 106.36
	PT (Sec)	14.46 ± 1.28	14.00 ± 0.84	13.22 ± 2.29	13.10 ± 0.46
	RET (%)	1.68 ± 0.18	2.28 ± 0.55	1.98 ± 1.11	1.44 ± 0.38
Male (n = 5)	WBC ($\times 10^9$/L)	6.99 ± 1.62	7.11 ± 1.03	6.71 ± 0.80	7.19 ± 2.02
	NE%	30.91 ± 1.38	30.45 ± 1.07	31.61 ± 0.89	30.62 ± 0.86
	LY%	62.11 ± 1.09	61.13 ± 1.58	61.20 ± 1.60	62.06 ± 0.52
	MO%	4.79 ± 0.44	5.89 ± 0.23$^{**▲▲}$	4.80 ± 0.76	5.29 ± 0.57
	RBC ($\times 10^{12}$/L)	7.49 ± 0.70	7.60 ± 0.24	7.98 ± 0.41	7.87 ± 0.56
	Hb (g/L)	135.60 ± 4.56	134.80 ± 6.10	139.60 ± 2.61	136.00 ± 5.10
	RDW (%)	17.24 ± 1.91	17.98 ± 1.01	17.60 ± 1.33	17.86 ± 1.14
	MCV (fL)	41.98 ± 2.24	40.56 ± 1.23	40.00 ± 1.30	40.10 ± 1.90
	MCH (pg)	18.14 ± 1.31	17.72 ± 0.58	17.52 ± 0.70	17.34 ± 0.97
	MCHC (g/L)	430.60 ± 9.07	439.20 ± 5.89	434.60 ± 8.90	434.00 ± 9.11
	PLT ($\times 10^9$/L)	808.40 ± 106.38	835.20 ± 111.16	818.80 ± 80.11	854.80 ± 84.20
	PT (Sec)	15.38 ± 1.19	15.56 ± 2.89	14.94 ± 2.00	12.42 ± 6.29
	RET (%)	1.52 ± 0.36	1.70 ± 0.47	1.46 ± 0.54	1.78 ± 0.42

Results were expressed as means ± SD.

$^{*/**}$ $p < 0.05/0.01$ was compared with the control group.

$^{\Delta}$ $p < 0.05$ was compared with the 50 mg/kg.bw group.

$^{▲/▲▲}$ $p < 0.05/0.01$ was compared with the 100 mg/kg.bw group.

Table S8 Serum chemistry values of rats treated with TA for 7 weeks

Indexes	Female (n = 5)				Male (n = 5)			
	Control	50 mg/kg.bw	100 mg/kg.bw	300 mg/kg.bw	Control	50 mg/kg.bw	100 mg/kg.bw	300 mg/kg.bw
ALT (U/L)	32.80 ± 12.64	30.20 ± 4.09	30.80 ± 10.59	25.00 ± 3.81	34.20 ± 5.63	34.00 ± 3.94	29.60 ± 3.13	32.60 ± 11.19
AST (U/L)	86.00 ± 12.59	82.80 ± 8.79	76.80 ± 17.21	74.20 ± 16.74	87.20 ± 14.84	88.80 ± 7.19	77.20 ± 14.72$^{\Delta}$	79.20 ± 6.30
ALP (U/L)	79.00 ± 17.65	78.60 ± 13.13	84.00 ± 12.02	75.00 ± 13.27	124.40 ± 6.54	117.60 ± 15.66	131.20 ± 8.79	119.00 ± 30.11
BUN (mmol/L)	5.02 ± 0.33	4.90 ± 0.42	4.40 ± 0.60	4.06 ± 0.51$^{**\Delta}$	4.12 ± 0.36	3.80 ± 0.43	3.94 ± 0.25	4.18 ± 0.41
CRE (μmol/L)	87.60 ± 12.78	94.20 ± 12.83	86.00 ± 11.22	88.40 ± 10.57	136.40 ± 58.45	104.40 ± 24.39	99.00 ± 32.95	121.60 ± 60.45
TP (g/L)	78.24 ± 10.69	75.78 ± 9.93	73.66 ± 5.96	77.06 ± 8.28	77.16 ± 3.81	73.16 ± 5.28	76.50 ± 17.93	91.88 ± 16.25
ALB (g/L)	31.40 ± 5.29	32.92 ± 3.53	33.18 ± 2.08	33.72 ± 3.06	32.80 ± 1.53	33.10 ± 0.39	32.30 ± 1.74	33.00 ± 2.67
Glu (mmol/L)	6.26 ± 0.36	6.42 ± 0.51	6.56 ± 0.40	7.54 ± 0.67$^{**\Delta\Delta}$▲▲	6.58 ± 0.69	6.22 ± 0.80	6.64 ± 1.19	6.80 ± 0.34
TBIL (μmol/L)	2.58 ± 1.27	3.42 ± 0.80	3.98 ± 0.84*	4.34 ± 0.22**	4.56 ± 2.30	4.78 ± 1.22	5.18 ± 2.25	5.52 ± 1.96
TC (mmol/L)	1.50 ± 0.17	1.57 ± 0.47	1.78 ± 0.19	1.77 ± 0.32	1.57 ± 0.36	1.30 ± 0.09	1.13 ± 0.14$^{**\nabla}$	1.44 ± 0.18
TG (mmol/L)	0.67 ± 0.20	0.60 ± 0.13	0.52 ± 0.24	0.60 ± 0.08	1.47 ± 0.54	1.17 ± 0.46	0.93 ± 0.29	1.06 ± 0.43
CK (U/L)	405.40 ± 214.36	378.40 ± 120.94	288.20 ± 123.62	434.40 ± 246.16	265.20 ± 37.88	247.40 ± 59.11	299.20 ± 131.85	274.20 ± 47.28
K^+ (mmol/L)	3.89 ± 0.40	4.22 ± 0.16	4.11 ± 0.37	4.25 ± 0.32	4.14 ± 0.35	4.09 ± 0.17	3.97 ± 0.36	4.05 ± 0.15
Na^+ (mmol/L)	139.26 ± 0.97	138.94 ± 0.98	139.46 ± 1.85	140.16 ± 1.37	140.48 ± 1.40	140.96 ± 1.20	139.22 ± 1.05	140.40 ± 1.25
Cl^- (mmol/L)	107.44 ± 0.72	108.42 ± 1.22	109.08 ± 1.25*∇	107.20 ± 1.00	107.00 ± 0.49	108.08 ± 1.72	107.70 ± 1.87	107.48 ± 1.46
iCa (mmol/L)	1.08 ± 0.03	1.14 ± 0.11	1.10 ± 0.12	1.07 ± 0.08	1.07 ± 0.05	1.06 ± 0.06	1.10 ± 0.07	1.09 ± 0.06

TCa （mmol/L）	2.11 ± 0.05	2.22 ± 0.21	2.15 ± 0.22	2.09 ± 0.15	2.08 ± 0.09	2.06 ± 0.11	2.14 ± 0.14	2.12 ± 0.11
pH	7.49 ± 0.004	7.50 ± 0.004	7.49 ± 0.01	7.50 ± 0.005	7.50 ± 0.004	7.49 ± 0.01	7.50 ± 0.009	7.50 ± 0.02

Results were expressed as means ± SD.

$^{*/**}$ $p < 0.05/0.01$ was compared with the control group.

$^{\Delta/\Delta\Delta}$ $p < 0.05/0.01$ was compared with the 50 mg/kg.bw group.

$^{\blacktriangle\blacktriangle}$$p < 0.01$ was compared with the 100 mg/kg.bw group.

$^{\nabla}$ $p < 0.05$ was compared with the 300 mg/kg.bw group.

Table S9 Serum chemistry values of rats treated with TA for 13 weeks

Indexes	Female (n = 10)				Male (n = 10)			
	Control	50 mg/kg.bw	100 mg/kg.bw	300 mg/kg.bw	Control	50 mg/kg.bw	100 mg/kg.bw	300 mg/kg.bw
ALT (U/L)	26.30 ± 4.30	30.50 ± 4.14	25.50 ± 3.27	27.50 ± 5.17	36.70 ± 5.70	33.40 ± 7.06	37.70 ± 4.22	29.30 ± 7.24$^{*\blacktriangle\blacktriangle}$
AST (U/L)	76.70 ± 12.55	79.50 ± 11.85	69.50 ± 8.15$^{\Delta}$	63.30 ± 9.63$^{**\Delta\Delta}$	80.70 ± 10.98	82.30 ± 11.88	85.00 ± 10.06	72.00 ± 13.55
ALP (U/L)	56.90 ± 12.62	61.30 ± 7.12	61.30 ± 7.90	75.20 ± 14.97$^{**\Delta\Delta\blacktriangle\blacktriangle}$	100.70 ± 9.26	96.00 ± 15.54	108.30 ± 16.01	104.50 ± 14.57
BUN (mmol/L)	3.85 ± 0.53	3.67 ± 0.40	3.85 ± 0.53	3.79 ± 0.56	4.04 ± 0.38	3.93 ± 0.56	3.80 ± 0.45	3.72 ± 0.70
CRE (μmol/L)	113.80 ± 34.65	114.50 ± 40.02	99.90 ± 12.93	100.70 ± 16.74	86.00 ± 7.04	90.90 ± 14.20	92.70 ± 20.28	92.00 ± 14.53
TP (g/L)	91.05 ± 12.70	92.92 ± 14.25	87.95 ± 16.12	93.81 ± 6.98	79.62 ± 9.02	82.98 ± 2.47	81.23 ± 6.35	81.63 ± 14.43
ALB (g/L)	35.58 ± 2.49	35.12 ± 1.70	35.53 ± 2.62	37.27 ± 2.98	32.67 ± 1.58	34.28 ± 0.81	33.47 ± 1.90	33.23 ± 2.14
Glu (mmol/L)	6.40 ± 0.64	6.55 ± 0.51	6.74 ± 0.68	8.11 ± 1.18$^{**\Delta\Delta\blacktriangle\blacktriangle}$	7.58 ± 1.84	6.95 ± 0.53	7.05 ± 0.48	6.92 ± 1.09
TBIL (μmol/L)	4.17 ± 1.83	4.37 ± 1.56	3.88 ± 1.01	4.16 ± 1.07	4.38 ± 1.09	4.26 ± 1.74	3.90 ± 1.31	4.49 ± 0.62
TC (mmol/L)	1.35 ± 0.18	1.43 ± 0.20	1.40 ± 0.17	1.56 ± 0.21	1.52 ± 0.22	1.57 ± 0.26	1.61 ± 0.25	1.74 ± 0.26
TG (mmol/L)	0.38 ± 0.19	0.43 ± 0.15	0.32 ± 0.15	0.52 ± 0.20	0.69 ± 0.27	0.99 ± 0.31*	1.04 ± 0.37*	1.01 ± 0.26*
CK (U/L)	210.10 ± 68.46	214.40 ± 77.52	203.80 ± 97.50	200.20 ± 86.45	175.00 ± 34.45	161.20 ± 50.25	168.00 ± 22.99	151.80 ± 64.51
K^+ (mmol/L)	3.42 ± 0.24	3.42 ± 0.21	3.49 ± 0.34	3.17 ± 0.37	3.28 ± 0.22	3.44 ± 0.26	3.44 ± 0.22	3.37 ± 0.21
Na^+ (mmol/L)	135.49 ± 1.10	135.43 ± 1.08	134.65 ± 0.71	134.59 ± 1.26	135.45 ± 0.96	135.15 ± 1.00	135.07 ± 1.35	135.06 ± 0.86
Cl^- (mmol/L)	102.59 ± 1.76	102.06 ± 2.17	102.14 ± 2.20	100.24 ± 0.93$^{**\Delta\blacktriangle}$	98.94 ± 2.44	98.84 ± 0.93	98.56 ± 1.25	98.14 ± 0.95
iCa (mmol/L)	1.03 ± 0.10	1.10 ± 0.09	1.08 ± 0.11	1.03 ± 0.05	0.99 ± 0.04	1.02 ± 0.03	1.03 ± 0.06	1.03 ± 0.06
TCa（mmol/L）	2.00 ± 0.20	2.14 ± 0.17	2.11 ± 0.22	2.01 ± 0.10	1.93 ± 0.08	1.99 ± 0.06	2.01 ± 0.12	2.01 ± 0.12
pH	7.52 ± 0.03	7.54 ± 0.03	7.53 ± 0.02	7.52 ± 0.01	7.51 ± 0.01	7.52 ± 0.01	7.52 ± 0.02	7.52 ± 0.01

Results were expressed as means ± SD.

$^{*/**}p <$ 0.05/0.01 was compared with the control group.

$^{\Delta/\Delta\Delta}$ $p < 0.05/0.01$ was compared with the 50 mg/kg.bw group.

$^{▲/▲▲}p < 0.05/0.01$ was compared with the 100 mg/kg.bw group.

Table S10 Serum chemistry values of rats after the 4-week recovery period

Indexes	Female (n = 5)				Male (n = 5)			
	Control	50 mg/kg.bw	100 mg/kg.bw	300 mg/kg.bw	Control	50 mg/kg.bw	100 mg/kg.bw	300 mg/kg.bw
ALT (U/L)	30.60 ± 6.02	34.00 ± 10.65	25.60 ± 5.18	26.60 ± 6.35	32.60 ± 2.07	35.00 ± 4.95	31.40 ± 4.39	36.40 ± 3.58
AST (U/L)	79.40 ± 12.34	71.80 ± 10.35	59.20 ± 7.01$^{**\Delta}$	61.00 ± 5.79**	84.00 ± 10.42	72.80 ± 15.99	73.60 ± 12.56	79.60 ± 7.76
ALP (U/L)	71.20 ± 36.19	70.20 ± 9.65	53.00 ± 8.34	93.80 ± 40.76	102.00 ± 11.73	105.00 ± 22.59	91.40 ± 9.86	77.60 ± 9.21$^{*\Delta\Delta}$
BUN (mmol/L)	3.80 ± 0.55	3.84 ± 0.55	3.68 ± 0.33	4.18 ± 0.50	4.16 ± 0.28	3.68 ± 0.26*	3.98 ± 0.41	3.24 ± 0.17$^{**\Delta\blacktriangle\blacktriangle}$
CRE (μmol/L)	88.20 ± 4.09	91.20 ± 10.47	90.60 ± 10.06	92.40 ± 16.83	85.40 ± 7.92	82.60 ± 2.70	80.60 ± 4.67	93.60 ± 16.32
TP (g/L)	94.64 ± 18.32	82.16 ± 4.44	82.64 ± 4.56	95.80 ± 15.54	76.14 ± 4.79	75.20 ± 3.83	81.16 ± 11.76	80.32 ± 15.16
ALB (g/L)	34.08 ± 0.08	34.14 ± 0.22	34.10 ± 0.22	34.06 ± 0.25	34.18 ± 0.19	34.30 ± 0.39	34.22 ± 0.16	34.30 ± 0.07
Glu (mmol/L)	6.58 ± 0.52	6.12 ± 0.79	6.64 ± 0.86	6.86 ± 0.39	6.54 ± 0.21	6.66 ± 0.80	6.36 ± 0.52	6.60 ± 0.67
TBIL (μmol/L)	2.84 ± 0.88	3.40 ± 1.43	3.62 ± 0.75	4.20 ± 2.14	4.14 ± 0.23	3.52 ± 0.54	3.34 ± 0.49	4.22 ± 0.78
TC (mmol/L)	2.04 ± 0.31	2.18 ± 0.22	2.10 ± 0.20	2.25 ± 0.30	1.93 ± 0.21	1.89 ± 0.18	1.73 ± 0.24	1.98 ± 0.42
TG (mmol/L)	0.70 ± 0.36	0.59 ± 0.44	0.57 ± 0.15	0.89 ± 0.78	1.50 ± 0.31	1.17 ± 0.53	0.89 ± 0.31	1.32 ± 0.66
CK (U/L)	231.40 ± 191.06	164.40 ± 60.74	122.40 ± 34.92	118.40 ± 34.52	241.00 ± 131.03	135.80 ± 9.83	216.60 ± 78.06	176.20 ± 66.16
K^+ (mmol/L)	3.79 ± 0.33	3.91 ± 0.18	3.70 ± 0.17	3.55 ± 0.19	3.96 ± 0.14	3.68 ± 0.26	3.81 ± 0.11	3.87 ± 0.10
Na^+ (mmol/L)	142.80 ± 1.26	142.20 ± 1.77	142.18 ± 1.63	143.52 ± 1.46	142.44 ± 1.98	143.10 ± 1.45	144.78 ± 1.55	142.94 ± 2.06
Cl^- (mmol/L)	116.56 ± 1.93	117.32 ± 1.66	118.58 ± 1.29	117.98 ± 2.10	115.76 ± 1.26	115.04 ± 5.42	116.42 ± 1.16	115.88 ± 2.49
iCa (mmol/L)	1.18 ± 0.07	1.15 ± 0.06	1.16 ± 0.06	1.13 ± 0.03	1.15 ± 0.09	1.14 ± 0.12	1.07 ± 0.07	1.14 ± 0.10
TCa（mmol/L）	2.30 ± 0.14	2.24 ± 0.12	2.27 ± 0.12	2.20 ± 0.06	2.23 ± 0.18	2.23 ± 0.22	2.08 ± 0.15	2.23 ± 0.20
pH	7.50 ± 0.008	7.50 ± 0.01	7.50 ± 0.01	7.50 ± 0.02	7.49 ± 0.02	7.50 ± 0.02	7.49 ± 0.01	7.50 ± 0.01

Results were expressed as means ± SD.

*/** $p < 0.05/0.01$ was compared with the control group.

Δ/ΔΔ $p < 0.05/0.01$ was compared with the 50 mg/kg.bw group.

▲▲$p < 0.05/0.01$ was compared with the 100 mg/kg.bw group.

Table S11 Organ weights and coefficients of rats treated with TA for 7 weeks

Items	Female (n = 5)				Male (n = 5)			
	Control	50 mg/kg.bw	100 mg/kg.bw	300 mg/kg.bw	Control	50 mg/kg.bw	100 mg/kg.bw	300 mg/kg.bw
Brain (g)	1.90 ± 0.04	1.94 ± 0.08	1.91 ± 0.03	1.86 ± 0.08	2.06 ± 0.07	2.04 ± 0.06	2.00 ± 0.04	2.03 ± 0.05
(%)	0.75 ± 0.06	0.75 ± 0.03	0.75 ± 0.02	0.80 ± 0.09	0.52 ± 0.04	0.56 ± 0.05	0.54 ± 0.03	0.55 ± 0.05
Heart (g)	0.95 ± 0.28	0.93 ± 0.07	0.93 ± 0.09	0.82 ± 0.06	1.42 ± 0.13	1.20 ± 0.13	1.33 ± 0.13	1.21 ± 0.14
(%)	0.37 ± 0.09	0.36 ± 0.03	0.36 ± 0.03	0.35 ± 0.02	0.35 ± 0.01	0.33 ± 0.04	0.36 ± 0.04	0.32 ± 0.03
Liver (g)	6.83 ± 0.70	7.20 ± 0.56	7.06 ± 0.67	7.83 ± 0.70	11.78 ± 1.92	10.31 ± 1.10	11.25 ± 0.86	12.29 ± 1.05
(%)	2.69 ± 0.19	2.78 ± 0.17	2.76 ± 0.18	3.37 ± 0.24$^{**\Delta\Delta\blacktriangle\blacktriangle}$	2.91 ± 0.18	2.79 ± 0.05	3.05 ± 0.22	3.31 ± 0.10$^{*\Delta\blacktriangle}$
Spleen (g)	0.79 ± 0.21	0.71 ± 0.05	0.67 ± 0.13	0.65 ± 0.13	0.88 ± 0.09	0.88 ± 0.20	0.78 ± 0.15	0.80 ± 0.11
(%)	0.31 ± 0.08	0.28 ± 0.02	0.26 ± 0.05	0.28 ± 0.04	0.22 ± 0.02	0.24 ± 0.05	0.21 ± 0.04	0.22 ± 0.04
Lung (g)	1.12 ± 0.06	1.13 ± 0.17	1.17 ± 0.10	1.24 ± 0.38	1.31 ± 0.08	1.44 ± 0.08	1.31 ± 0.13	1.40 ± 0.22
(%)	0.44 ± 0.04	0.43 ± 0.04	0.46 ± 0.02	0.53 ± 0.14	0.33 ± 0.04	0.39 ± 0.02	0.35 ± 0.02	0.38 ± 0.06
Kidney (g)	1.59 ± 0.17	1.66 ± 0.05	1.57 ± 0.06	1.63 ± 0.17	2.62 ± 0.34	2.42 ± 0.18	2.47 ± 0.10	2.45 ± 0.17
(%)	0.63 ± 0.04	0.64 ± 0.04	0.62 ± 0.03	0.70 ± 0.01$^{**\Delta\Delta\blacktriangle\blacktriangle}$	0.65 ± 0.03	0.66 ± 0.02	0.67 ± 0.02	0.66 ± 0.05
Adrenal gland (g)	0.071 ± 0.003	0.073 ± 0.006	0.071 ± 0.010	0.077 ± 0.010	0.048 ± 0.004	0.045 ± 0.007	0.049 ± 0.004	0.042 ± 0.004
(%)	0.028 ± 0.002	0.028 ± 0.002	0.028 ± 0.004	0.033 ± 0.004	0.012 ± 0.001	0.012 ± 0.002	0.013 ± 0.002	0.011 ± 0.002
Thymus (g)	0.38 ± 0.07	0.43 ± 0.14	0.43 ± 0.09	0.39 ± 0.06	0.44 ± 0.14	0.36 ± 0.07	0.36 ± 0.13	0.39 ± 0.08
(%)	0.15 ± 0.02	0.16 ± 0.05	0.17 ± 0.04	0.17 ± 0.03	0.11 ± 0.03	0.10 ± 0.02	0.10 ± 0.04	0.10 ± 0.02
Uterus/ Testis (g)	0.40 ± 0.07	0.40 ± 0.10	0.50 ± 0.18	0.64 ± 0.29	2.72 ± 0.45	2.77 ± 0.18	2.75 ± 0.10	2.66 ± 0.26
(%)	0.16 ± 0.02	0.16 ± 0.04	0.20 ± 0.07	0.27 ± 0.12	0.68 ± 0.14	0.76 ± 0.07	0.75 ± 0.04	0.72 ± 0.05
Ovary/Epididymis (g)	0.112 ± 0.007	0.114 ± 0.010	0.108 ± 0.010	0.101 ± 0.020	0.95 ± 0.12	0.97 ± 0.06	0.96 ± 0.06	0.96 ± 0.06
(%)	0.044 ± 0.006	0.044 ± 0.003	0.042 ± 0.005	0.043 ± 0.006	0.24 ± 0.04	0.26 ± 0.03	0.26 ± 0.02	0.26 ± 0.02

Results were expressed as means ± SD.

*/** $p < 0.05/0.01$ was compared with the control group.

Δ/ΔΔ $p < 0.05/0.01$ was compared with the 50 mg/kg.bw group.

▲/▲▲ $p < 0.05/0.01$ was compared with the 100 mg/kg.bw group.

♀, uterus, ovary; ♂, testis, epididymis.

Table S12 Organ weights and coefficients of rats treated with TA for 13 weeks

Items	Female (n = 10)				Male (n = 10)			
	Control	50 mg/kg.bw	100 mg/kg.bw	300 mg/kg.bw	Control	50 mg/kg.bw	100 mg/kg.bw	300 mg/kg.bw
Brain (g)	1.99 ± 0.07	2.00 ± 0.08	1.97 ± 0.06	1.95 ± 0.06	2.18 ± 0.08	2.16 ± 0.08	2.16 ± 0.06	2.16 ± 0.09
(%)	0.70 ± 0.06	0.70 ± 0.04	0.69 ± 0.06	0.72 ± 0.07	0.44 ± 0.04	0.45 ± 0.04	0.45 ± 0.02	0.48 ± 0.03**Δ▲
Heart (g)	0.96 ± 0.13	1.02 ± 0.15	0.95 ± 0.08	0.92 ± 0.10	1.50 ± 0.13	1.43 ± 0.12	1.48 ± 0.15	1.41 ± 0.09
(%)	0.33 ± 0.03	0.35 ± 0.03	0.33 ± 0.03	0.33 ± 0.03	0.30 ± 0.02	0.30 ± 0.02	0.31 ± 0.03	0.32 ± 0.02
Liver (g)	7.63 ± 0.76	7.49 ± 0.72	7.74 ± 0.86	9.68 ± 1.26**ΔΔ▲▲	12.64 ± 1.01	13.09 ± 1.02	13.60 ± 0.93*	14.70 ± 0.65**ΔΔ▲
(%)	2.66 ± 0.11	2.59 ± 0.15	2.72 ± 0.20	3.52 ± 0.26*Δ▲	2.56 ± 0.10	2.73 ± 0.14**	2.85 ± 0.10**Δ	3.31 ± 0.12**ΔΔ▲▲
Spleen (g)	0.64 ± 0.09	0.68 ± 0.10	0.73 ± 0.14	0.58 ± 0.11Δ▲▲	0.90 ± 0.17	0.88 ± 0.12	0.88 ± 0.17	0.84 ± 0.11
(%)	0.23 ± 0.04	0.24 ± 0.02	0.26 ± 0.06	0.21 ± 0.03	0.18 ± 0.03	0.18 ± 0.02	0.18 ± 0.04	0.19 ± 0.02
Lung (g)	1.16 ± 0.21	1.13 ± 0.13	1.09 ± 0.04	1.07 ± 0.07	1.52 ± 0.18	1.40 ± 0.10*	1.46 ± 0.13	1.33 ± 0.05**▲
(%)	0.40 ± 0.06	0.39 ± 0.03	0.38 ± 0.02	0.39 ± 0.02	0.31 ± 0.04	0.29 ± 0.02	0.31 ± 0.02	0.30 ± 0.02
Kidney (g)	1.76 ± 0.15	1.74 ± 0.16	1.72 ± 0.12	1.90 ± 0.24	2.84 ± 0.26	2.82 ± 0.17	2.79 ± 0.17	2.77 ± 0.28
(%)	0.62 ± 0.06	0.60 ± 0.04	0.60 ± 0.04	0.69 ± 0.05**ΔΔ▲▲	0.58 ± 0.04	0.59 ± 0.04	0.58 ± 0.03	0.62 ± 0.06
Adrenal (g)	0.077 ± 0.010	0.072 ± 0.010	0.074 ± 0.007	0.073 ± 0.008	0.059 ± 0.007	0.053 ± 0.008	0.054 ± 0.009	0.050 ± 0.008
(%)	0.027 ± 0.004	0.025 ± 0.003	0.026 ± 0.003	0.027 ± 0.002	0.012 ± 0.002	0.011 ± 0.002	0.011 ± 0.002	0.011 ± 0.002
Thymus (g)	0.29 ± 0.04	0.29 ± 0.05	0.28 ± 0.05	0.25 ± 0.04	0.28 ± 0.06	0.24 ± 0.05	0.26 ± 0.03	0.26 ± 0.05
(%)	0.10 ± 0.01	0.10 ± 0.01	0.10 ± 0.02	0.09 ± 0.01	0.057 ± 0.011	0.051 ± 0.010	0.054 ± 0.059	0.058 ± 0.012
Uterus/ Testis (g)	0.55 ± 0.07	0.60 ± 0.16	0.59 ± 0.20	0.55 ± 0.11	3.13 ± 0.19	3.00 ± 0.25	2.87 ± 0.12**	2.99 ± 0.20
(%)	0.19 ± 0.03	0.20 ± 0.05	0.21 ± 0.08	0.20 ± 0.05	0.64 ± 0.06	0.63 ± 0.04▽	0.60 ± 0.04▽▽	0.67 ± 0.06
Ovary/Epididymis (g)	0.115 ± 0.015	0.112 ± 0.012	0.114 ± 0.006	0.108 ± 0.010	1.21 ± 0.09	1.13 ± 0.08	1.23 ± 0.23	1.14 ± 0.07
(%)	0.040 ± 0.006	0.039 ± 0.004	0.040 ± 0.004	0.040 ± 0.004	0.24 ± 0.03	0.24 ± 0.02	0.26 ± 0.07	0.26 ± 0.01

Results were expressed as means ± SD.

*/** $p < 0.05/0.01$ was compared with the control group.

Δ/ΔΔ $p < 0.05/0.01$ was compared with the 50 mg/kg.bw group.

▲/▲▲ $p < 0.05/0.01$ was compared with the 100 mg/kg.bw group.

♀, uterus, ovary; ♂, testis, epididymis.

Table S13 Organ weights and coefficients of rats after the 4-week recovery period

Items	Female (n = 10)				Male (n = 10)			
	Control	50 mg/kg.bw	100 mg/kg.bw	300 mg/kg.bw	Control	50 mg/kg.bw	100 mg/kg.bw	300 mg/kg.bw
Brain (g)	1.92 ± 0.06	2.01 ± 0.04	2.00 ± 0.07	1.96 ± 0.08	2.20 ± 0.07	2.11 ± 0.03**	2.16 ± 0.05	2.09 ± 0.02**▲
(%)	0.60 ± 0.07	0.66 ± 0.04	0.66 ± 0.04	0.66 ± 0.07	0.39 ± 0.05	0.43 ± 0.02	0.41 ± 0.02	0.40 ± 0.03
Heart (g)	1.15 ± 0.36	1.14 ± 0.34	0.96 ± 0.07	0.93 ± 0.07	1.71 ± 0.23	1.43 ± 0.21	1.62 ± 0.27	1.54 ± 0.22
(%)	0.35 ± 0.06	0.37 ± 0.10	0.31 ± 0.02	0.31 ± 0.04	0.30 ± 0.02	0.29 ± 0.03	0.31 ± 0.03	0.29 ± 0.03
Liver (g)	8.11 ± 1.08	8.20 ± 1.05	7.83 ± 0.96	8.56 ± 1.51	14.70 ± 1.84	12.32 ± 0.81	13.97 ± 1.63	14.30 ± 2.24
(%)	2.52 ± 0.23	2.71 ± 0.36	2.56 ± 0.20	2.86 ± 0.37	2.60 ± 0.08	2.51 ± 0.14	2.66 ± 0.26	2.69 ± 0.26
Spleen (g)	0.63 ± 0.03	0.73 ± 0.11	0.65 ± 0.06	0.54 ± 0.04*∆▲	0.97 ± 0.19	0.89 ± 0.16	0.86 ± 0.09	0.94 ± 0.19
(%)	0.20 ± 0.02	0.24 ± 0.03*▽▽	0.21 ± 0.02	0.18 ± 0.02	0.17 ± 0.04	0.18 ± 0.03	0.16 ± 0.01	0.18 ± 0.02
Lung (g)	1.24 ± 0.15	1.24 ± 0.13	1.10 ± 0.04	1.08 ± 0.07	1.58 ± 0.23	1.40 ± 0.09	1.50 ± 0.17	1.49 ± 0.07
(%)	0.38 ± 0.01	0.41 ± 0.04	0.36 ± 0.03	0.36 ± 0.03	0.28 ± 0.02	0.28 ± 0.02	0.29 ± 0.02	0.28 ± 0.01
Kidney (g)	1.81 ± 0.23	1.82 ± 0.14	1.80 ± 0.20	1.80 ± 0.17	3.11 ± 0.40	2.76 ± 0.34	3.12 ± 0.35	3.14 ± 0.41
(%)	0.56 ± 0.06	0.60 ± 0.05	0.59 ± 0.06	0.60 ± 0.07	0.55 ± 0.05	0.56 ± 0.04	0.60 ± 0.06	0.59 ± 0.05
Adrenal (g)	0.067 ± 0.006	0.071 ± 0.011	0.069 ± 0.010	0.066 ± 0.005	0.055 ± 0.008	0.051 ± 0.008	0.050 ± 0.008	0.052 ± 0.009
(%)	0.021 ± 0.004	0.024 ± 0.004	0.023 ± 0.004	0.022 ± 0.002	0.010 ± 0.0005	0.010 ± 0.002	0.010 ± 0.001	0.010 ± 0.002
Thymus (g)	0.22 ± 0.07	0.24 ± 0.02	0.28 ± 0.10	0.20 ± 0.06	0.22 ± 0.06	0.24 ± 0.07	0.22 ± 0.08	0.24 ± 0.05
(%)	0.068 ± 0.018	0.080 ± 0.006	0.091 ± 0.030	0.067 ± 0.025	0.04 ± 0.01	0.05 ± 0.01	0.04 ± 0.01	0.04 ± 0.01
Uterus/ Testis (g)	0.56 ± 0.12	0.54 ± 0.21	0.59 ± 0.11	0.71 ± 0.29	3.08 ± 0.41	2.88 ± 0.39	3.03 ± 0.18	3.05 ± 0.19
(%)	0.18 ± 0.06	0.18 ± 0.06	0.19 ± 0.03	0.24 ± 0.10	0.55 ± 0.05	0.58 ± 0.05	0.58 ± 0.02	0.58 ± 0.03
Ovary/Epididymis (g)	0.115 ± 0.011	0.116 ± 0.011	0.110 ± 0.007	0.116 ± 0.004	1.19 ± 0.15	1.11 ± 0.11	1.20 ± 0.14	1.48 ± 0.78
(%)	0.036 ± 0.007	0.038 ± 0.003	0.036 ± 0.004	0.039 ± 0.003	0.21 ± 0.04	0.22 ± 0.02	0.23 ± 0.02	0.28 ± 0.15

Results were expressed as means ± SD.

*/** $p < 0.05/0.01$ was compared with the control group.

△ $p < 0.05$ was compared with the 50 mg/kg.bw group.

▲ $p < 0.05$ was compared with the 100 mg/kg.bw group.

▽▽ $p < 0.01$ was compared with the 300 mg/kg.bw group.

♀, uterus, ovary; ♂, testis, epididymis.

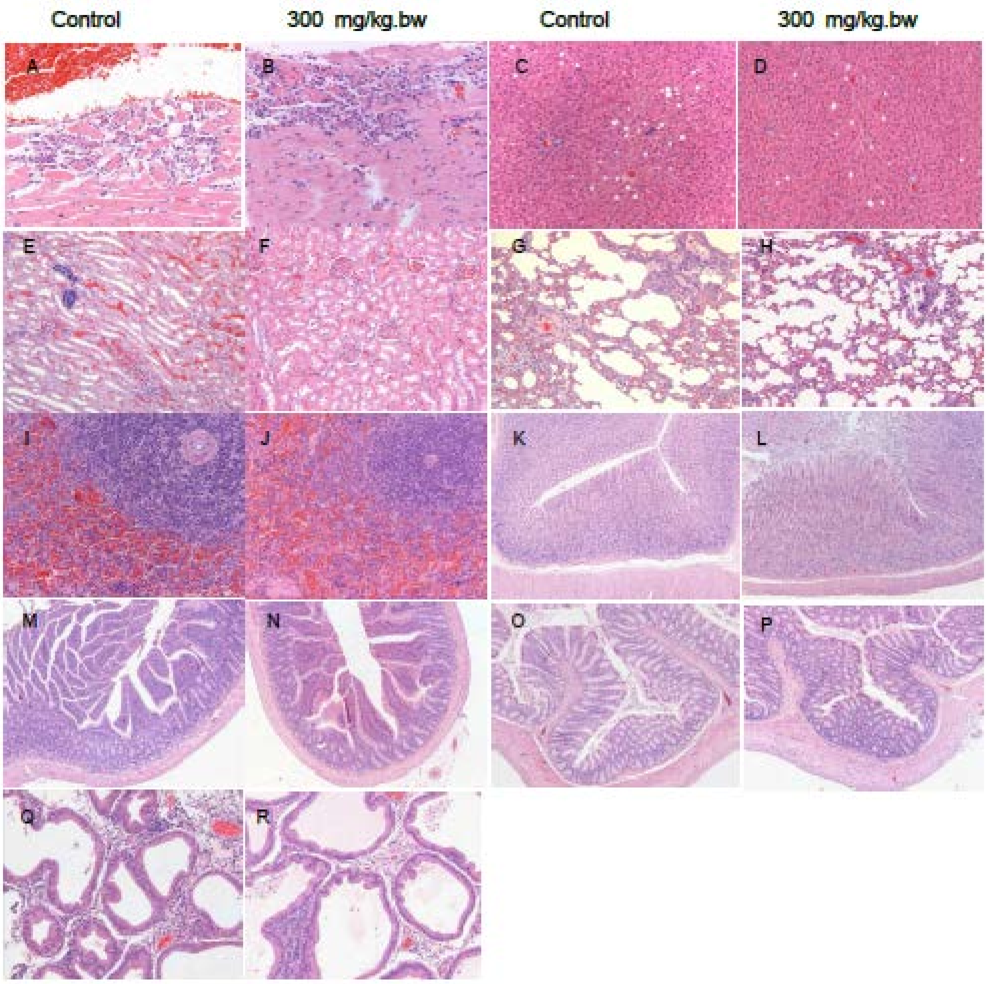

Fig. S1. Representative sections of rats in respect of 7 weeks treatment demonstrating histopathology

A and **B** represented control and 300 mg/kg.bw rat hearts showing local myocarditis (× 20 magnification).

C and **D** denoted control and 300 mg/kg.bw rat livers illuminating hepatocytes punctate necrotic and scattered cellular steatosis (× 10 magnification).

E and **F** meant control and 300 mg/kg.bw rat kidneys, which demonstrated calcium salt deposited locally at the junction of renal cortex and medulla in the control group and normal kidney architecture in 300 mg/kg.bw (× 10 magnification).

G and **H** represented control and 300 mg/kg.bw rat lungs showing local interstitial pneumonia (× 10 magnification).

I and **J** denoted the normal spleen architectures of rats in control and 300 mg/kg.bw group (× 20 magnification).

K and **L** represented the normal stomach architectures of rats in control and 300 mg/kg.bw group (× 5 magnification).

M and **N** showed the normal small intestine architectures of rats in control and 300 mg/kg.bw group (× 5 magnification).

O and **P** expressed the normal colon architectures of rats in control and 300 mg/kg.bw group (× 5 magnification).

Q and **R** represented control and 300 mg/kg.bw rat prostate glands accompanying by local prostatitis (× 10 magnification).

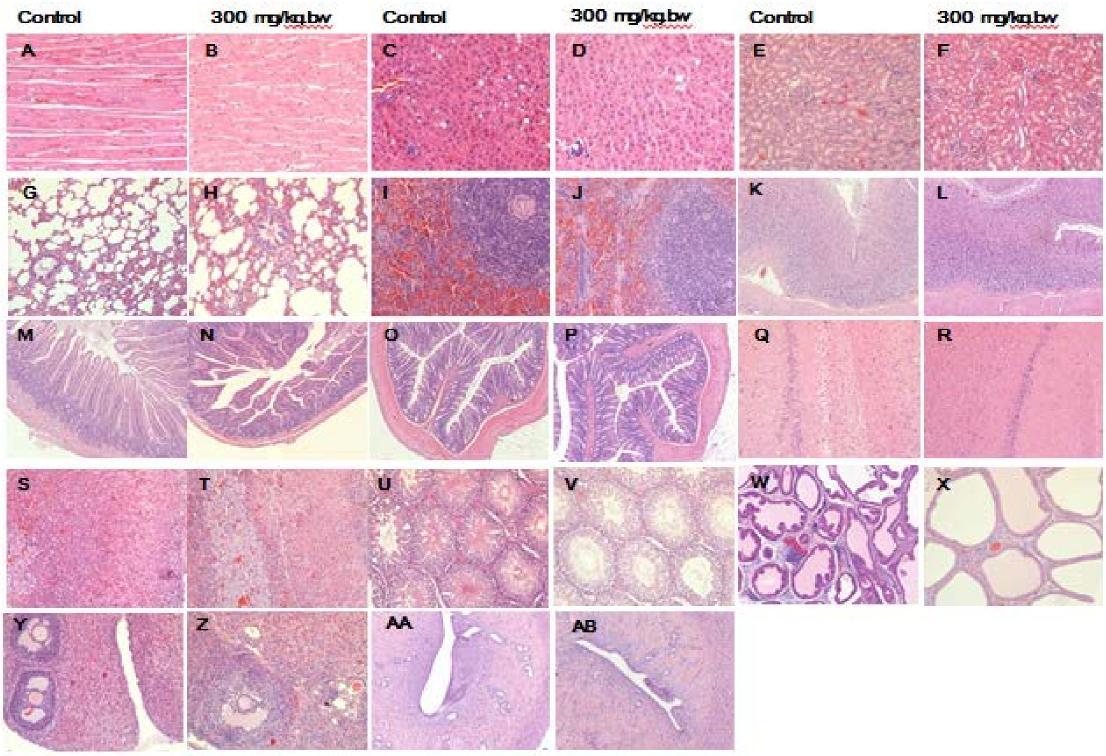

Fig. S2. Representative sections of rats in respect of 13 weeks treatment demonstrating histopathology

A and **B** the normal heart architectures of rats in control and 300 mg/kg.bw group (× 20 magnification).

C and **D** denoted control and 300 mg/kg.bw rat livers illuminating hepatocytes punctate necrotic and scattered cellular steatosis (× 10 magnification).

E and **F** meant the normal kidney architectures of rats in control and 300 mg/kg.bw group (× 10 magnification).

G and **H** represented control and 300 mg/kg.bw rat lungs showing local interstitial pneumonia (× 10 magnification).

I and **J** denoted the normal spleen architectures of rats in control and 300 mg/kg.bw group (× 20 magnification).

K and **L** represented the normal stomach architectures of rats in control and 300 mg/kg.bw group

(× 5 magnification).

M and **N** showed the normal small intestine architectures of rats in control and 300 mg/kg.bw group (× 5 magnification).

O and **P** expressed the normal colon architectures of rats in control and 300 mg/kg.bw group (× 5 magnification).

Q and **R** meant the normal brain architectures of rats in control and 300 mg/kg.bw group (× 10 magnification).

S and **T** denoted the normal adrenal gland architectures of rats in control and 300 mg/kg.bw group (× 10 magnification).

U and **V** represented the normal testis architectures of rats in control and 300 mg/kg.bw group (× 10 magnification).

W and **X** denoted control and 300 mg/kg.bw rat prostate glands accompanying by local prostatitis (× 10 magnification).

Y and **Z** showed the normal ovary architectures of rats in control and 300 mg/kg.bw group (× 10 magnification).

AA and **AB** expressed the normal uterus architectures of rats in control and 300 mg/kg.bw group (× 5 magnification).

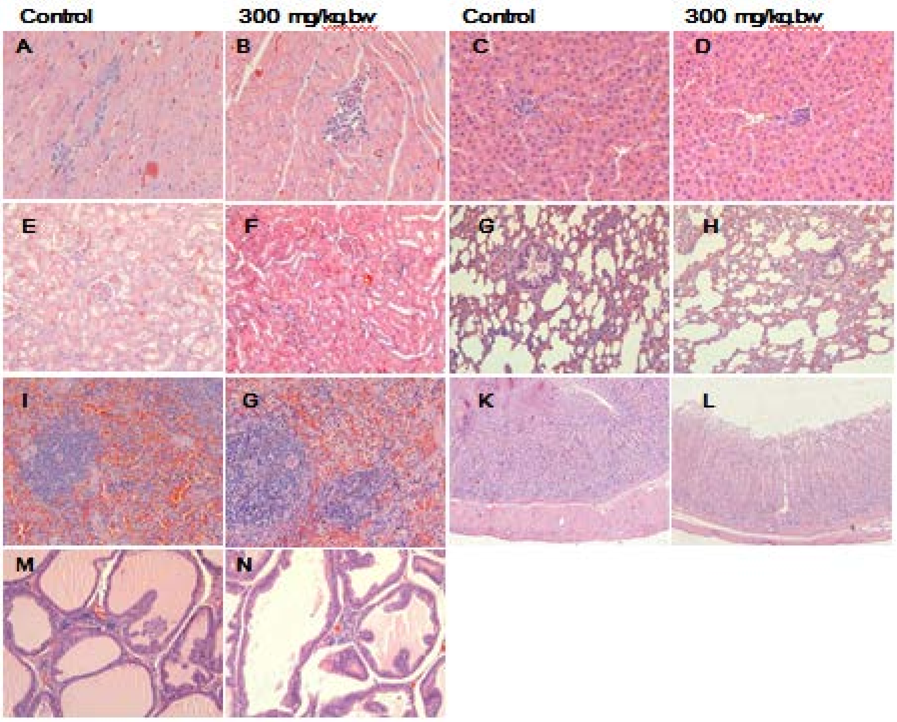

Fig. S3. Representative sections of rats in the recovery period demonstrating histopathology

A and **B** represented control and 300 mg/kg.bw rat hearts showing local myocarditis (× 20 magnification).

C and **D** denoted control and 300 mg/kg.bw rat livers illuminating hepatocytes punctate necrotic and scattered cellular steatosis (× 10 magnification).

E and **F** meant the normal kidney architectures of rats in control and 300 mg/kg.bw group (× 10 magnification).

G and **H** represented control and 300 mg/kg.bw rat lungs showing local interstitial pneumonia (× 10 magnification).

I and **J** denoted the normal spleen architectures of rats in control and 300 mg/kg.bw group (× 20 magnification).

K and **L** represented the normal stomach architectures of rats in control and 300 mg/kg.bw group (× 5 magnification).

M and **N** showed control and 300 mg/kg.bw rat prostate glands accompanying by local prostatitis (× 10 magnification).

References

(S1). Center for Drug Evaluation and Research. Guidance for Industry: Bioanalytical Method Validation. http://www.fda.gov/.
